# Isolation of N-Fixing Bacteria from Warm-Season Pasture Grasses and the Evaluation of Nitrogen Effects on the Bacterial Communities Present in *Cenchrus clandestinus*

**DOI:** 10.3390/microorganisms14040786

**Published:** 2026-03-30

**Authors:** Rens R. T. van Essen, Jatinder Kaur, Tongda Li, Tim I. Sawbridge

**Affiliations:** 1 Department of Energy, Environment and Climate Action, Agriculture Victoria, AgriBio, Centre for AgriBioscience, Bundoora, VIC 3083, Australia; jatinder.kaur@agriculture.vic.gov.au (J.K.); tongda.li@agriculture.vic.gov.au (T.L.); 2 School of Applied Systems Biology, La Trobe University, Bundoora, VIC 3083, Australia

**Keywords:** nitrogen fixation, warm-season pasture grass seeds, *Cenchrus clandestinus*, metagenomics

## Abstract

Nitrogen is essential for plant growth. Reliance on synthetic nitrogen fertilisers, however, is costly and contributes to soil degradation. Utilising nitrogen-fixing bacteria as biofertilisers may offer a sustainable alternative, reducing fertiliser costs and environmental impact. In this study, we attempted to isolate nitrogen-fixing bacteria from 14 seed batches of warm-season pasture grasses and successfully isolated bacteria from three of these batches. Whole genome sequencing confirmed the presence of the *nif* operon within all three isolates. Two seed batches of *Cenchrus clandestinus* (Hochst. ex Chiov.) Morrone from which *nif*-containing bacteria were isolated, along with two ‘*nif*’-negative *C. clandestinus* seed batches, were used in nitrogen-limiting growth assays. This was done to evaluate the effect of the presence of seed-associated nitrogen-fixing bacteria within a seed batch on nitrogen-limited plant growth and the associated plant microbiome composition, using 16S amplicon sequencing of root and shoot samples. Initial plant growth assays revealed significantly reduced root length between plants grown from seed batches harbouring nitrogen-fixing bacteria and those without, under limiting nitrogen availability, but no resulting shoot biomass reduction was observed. The plant microbiomes of these *nif*-positive seed batches were also statistically similar to each other, compared to the *nif*-negative seed batch plants. Plant microbiomes of all four *C. clandestinus* seed batches were significantly different from their original seed microbiomes, showing shifts in community composition. This study demonstrates the presence of potential nitrogen-fixing bacteria associated with warm-season pasture grass seeds at low abundance and reveals differences in plant-associated bacterial community composition between seed batches harbouring and lacking these bacteria.

## 1. Introduction

With a continuously growing global human population, there is an increasing demand for agricultural products [1]. To keep up with the increasing food demands, farmers have been using chemicals to increase crop yield, especially when growing in nutrient-deficient soils [2]. However, synthetic fertilisers are having a large impact on the environment around the farms, with excess chemicals contaminating the soil, air, and water sources around the farms [3]. As a result, soil health degrades, and this has a long-term effect on crop productivity [4]. In addition, the prices of synthetic fertilisers and, in particular, nitrogen have increased rapidly. These issues have led to an increase in popularity for biofertilisers, which are able to reduce agricultural chemical costs, regenerate soil health, and reduce environmental contamination [5]. The market value of biological products was worth USD 2.31 billion for biofertilisers and USD 7.54 billion for bioprotectants in 2023 and is projected to grow to USD 4.77 billion and USD 28.61 billion, respectively, by 2032 [6,7]. Biological products have the potential to protect the crops against pests and pathogens, increase the level of nitrogen through nitrogen fixation, increase the level of phosphate through phosphate solubilisation, and increase the availability of potassium through the breakdown of insoluble potassium [8]. Nitrogen in particular is one of the most vital nutrients for plant growth and crop productivity [9]. Nitrogen is vital for plant growth as it is a structural part of amino acids, chlorophyll, nucleic acids, and ATP [10]. Additionally, nitrogen plays a crucial role in carbon and nitrogen metabolisms, photosynthesis, and protein production [11]. However, usable nitrogen is often in short supply, limiting plant growth [12]. This highlights the importance of nitrogen for plant growth and explains why many farmers rely heavily on nitrogen fertilisation. Consequently, finding environmentally friendly alternatives to synthetic fertilisers is of great importance.

The seed microbiota is vital for the seed to transition to a seedling and the resulting plant fitness [13]. Seed microbiota has been found to influence plant health, yield, and resilience against environmental stresses [14]. Furthermore, the plant microbiome contains a highly diverse selection of microbes selected through environmental pressures and through vertical transmission [13]. This diverse selection of plant beneficial microbes is an important source for the discovery of microbes that could reduce the synthetic fertilisers commonly used in agriculture and provide a sustainable alternative [15]. Bacteria associated with seeds could have an advantage in the application of these microbes to seeds to support early stages of plant growth, which is vital to long-term plant health [16]. Bacteria found within the seed have been shown to harbour a range of beneficial traits, including biological nitrogen fixation [14].

Biological nitrogen fixation relies on bacteria in the rhizosphere, typically found in nodules on the root systems of legume plants [17]. Even though nitrogen is one of the most abundant elements in the atmosphere, plants are unable to utilise this form of nitrogen (N_2_) directly [10]. Nitrogen-fixing bacteria capture this atmospheric nitrogen and convert N_2_ into ammonia (NH_3_), which is suitable for plant use [18]. These bacteria are found to play a key role in the reduction in synthetic N fertiliser use and, as a result, are at the core of effectively reducing nitrous oxide (N_2_O) emissions [19]. At the core of this process is the *nif* operon, regulating and expressing nitrogen fixation genes under low nitrogen conditions [20]. Some nitrogen-fixing bacteria express nod factors, which signal the plants to create nodules on the roots, which the bacteria colonise and perform nitrogen fixation in exchange for carbon provided by the plants [21]. However, nodulation is a rare trait expressed mainly in plants within the legume family with very few exceptions [22]. For non-leguminous plants, diazotrophic bacteria perform nitrogen fixation in the soil and around roots without forming a specific symbiotic relationship with the plant [23]. Many important crops would benefit greatly from incorporating free-living nitrogen-fixing bacteria to improve plant growth at different stages when grown under nitrogen-limited conditions [9]. Targeting N-fixing bacterial isolation, warm-season pasture grasses are of interest due to their ability to grow well under nitrogen-deficient conditions, as these grasses contain the NADP-malic enzyme (ME), allowing them to rely less on available nitrogen compared to containing NAD-ME [24]. Consequently, these grasses grow well in soil with less available nitrogen, which is a prime environment for N-fixing bacteria to colonise and possibly transfer to the next generation of seeds [25]. In addition, warm-season pasture grasses are becoming of increasing interest for the global dairy industry due to increasing temperatures around the world [26]. Understanding how to utilise the microbiome of these favourable grasses could be very beneficial for the future of the dairy industry. In an initial study investigating the seed microbiome of warm-season pasture grasses, attempts to isolate N-fixing bacteria with a non-selective medium were unsuccessful [26]. A study in the cool-season pasture grass perennial ryegrass demonstrated that pasture grass seeds could harbour diazotrophic bacteria at low abundance [27]. Perennial ryegrass is a C3-grass, while the warm-season pasture grasses used within this study are C4-grasses, and we hypothesized these grasses could also harbour diazotrophic bacteria [28].

The aim of this study was to assess whether it was possible to isolate potential nitrogen-fixing bacteria from warm-season pasture grass seeds using nitrogen-deficient selective media. In addition, we investigated the effects of nitrogen on *Cenchrus clandestinus* (Hochst. ex Chiov.) Morrone seed batches harbouring potential N-fixing bacteria; here, the effects on plant growth and the effects on associated root and shoot bacterial communities were assessed and directly compared to two *C. clandestinus* seed batches, which did not harbour potential N-fixing bacteria. Understanding how plants and associated bacterial communities behave under different nitrogen conditions could give insights into how plants and their associated bacterial communities adapt to different nitrogen pressures. This study could lead to a better understanding of how to utilise these bacteria to aid plant growth.

## 2. Materials and Methods

### 2.1. Warm-Season Pasture Grass Seeds

Three different genera of warm-season grass seeds were sourced from four different distributors (Table 1). The seed batches included cv. Whittet of *Cenchrus clandestinus* obtained from McKays Seeds (Australia), Anco Seeds (Melbourne, Australia), and Williams Group Australia (Murwillumbah, Australia), with cv Acacia plateau also obtained from the Williams Group. Six cultivars of *Chloris gayana* Kunth (Katambora, Mariner, Tolgar, Endura, Callide, and Excel) were obtained from McKays Seeds, Barenbrug (Melbourne, Australia), and Williams Group Australia. Two *Paspalum* species were acquired (*Paspalum dilatatum* Poir. and *Paspalum notatum* Flüggé) from Williams Group, Australia, and McKays Seeds. The seed-associated microbiomes of these seed batches have been described in van Essen et al. 2025 [26]. The microbiomes for *C. clandestinus* from this seed-based study were used for comparison to the plant microbiomes generated in this current study from the same seed batches.

### 2.2. Isolation of Nitrogen Fixing Bacteria

For the isolation of potential nitrogen-fixing bacteria from the 14 seed batches described in Table 1, approximately 1000 seeds per batch were used. The seeds were washed by submerging them in sterile distilled water (SDW). The washed seeds were subsequently ground and submerged in 30 mL Burk’s N-free media (MgSO_4_, 0.2 g/L; K_2_HPO_4_, 0.8 g/L; KH_2_PO_4_, 0.2 g/L; CaSO_4_, 0.13 g/L; FeCl_3_, 0.00145 g/L; Na_2_MoO_4_, 0.000253 g/L; sucrose, 20 g/L). These seed-based cultures were grown at a temperature of 28 °C while rotating at 200 revolutions per minute (RPM) for 48 h. These cultures were used to create three serial dilutions (10^−1^, 10^−2^, and 10^−3^) in fresh Burk’s media. From these three dilutions, 50 μL was subsequently added to 5 mL of semi-solid Burk’s media (containing 1.6 g/L of agar) by pipetting the volume roughly 1 cm under the semi-solid media surface. These semi-solid cultures were incubated for five days at 28 °C. If microbial growth developed under the surface of the media, some of this growth was used to create five serial dilutions (10^−1^, 10^−2^, 10^−3^, 10^−4^, and 10^−5^) in fresh Burk’s media, which were source cultures for the plates. From each of these dilutions, 50 μL was also transferred to solid Burk’s media plates containing 15 g/L of agar. These plates were incubated for five days at 28 °C until single colonies could be picked. Single colonies were picked and streaked on solid Burk’s media plates to create clean, single, pure isolate cultures.

### 2.3. NifH PCR

Source cultures and single colonies from the previous section were used as an input of DNA for a *nif*H PCR. For the source cultures, 20 μL of each starting culture was used and incubated at 99 °C for 10 min in a thermocycler. Single colonies were dissolved in 50 μL SDW, from which 20 μL was added to a tube for incubation at 99 °C for 10 min in a thermocycler. The samples were centrifuged at 4000 RPM for 5 min, and 5 µL of the supernatant was used as a template for the PCR. The PCR contained OneTaq 2X Master Mix with Standard Buffer (NEBiolabs, Cat# M0482S) (Ipswich, MA, USA) and the primers, IGK3 (Forward, 5′-GCIWTHTAYGGIAARGGIGGIATHGGIA-3′) and DVV (Reverse, 5′-ATIGCRAAICCICCRCAIACIACRTC-3′), which target the *nif*H gene of the *nif* operon [29]. The final concentration of each primer was 10 μM. The PCR programme used included an initial denaturation step at 94 °C for 30 s, followed by 30 cycles of denaturation at 94 °C for 30 s, annealing at 58 °C for 60 s, and extension at 68 °C for 60 s, followed by a final extension step at 68 °C for 5 min. The PCR was visualised through High Sensitivity DNA (HSD) 1000 ScreenTape on TapeStation (Agilent technology cat# 5067-5584) (Santa Clara, CA, USA) to ensure that amplification of the target had occurred.

### 2.4. Whole Genome Sequencing

Whole Genome Sequencing (WGS) was performed on isolates that showed amplification of the *nif*H gene. From these isolates, DNA was extracted using a ZymoBIOMICS DNA Miniprep Kit (Zymo Research, cat# D4300T) (Orange, CA, USA) following the protocol supplied with the kit. Sequencing of the extracted DNA was done on an Oxford Nanopore Technology (ONT) platform using ONT Native Barcoding Kit 24 V14 (SQK-NBD114.24) (Oxford, UK) with some modifications. For the initial DNA repair and end-prep, the volumes for all the components were doubled except for the DNA volume. The subsequent cleaning step was performed using Promega ProNex Size-Selective Purification system beads (ratio of 1.5× (*v*/*v*)) (Cat# NG2001). The native barcode ligation was modified by adding an extra 10 μL End-prepped gDNA to a total volume of 17.5 μL. The Blunt/TA Ligase Master Mix was increased by 10 μL to a total volume of 20 μL. After the ligation, a second clean-up was done using the ProNex Size-Selective Purification system beads (ratio of 1.5× (*v*/*v*)). Priming and loading the SpotON flow cell were done following the original ONT protocol using library loading beads. The library was sequenced on an ONT MinION Mk1C system (Oxford Nanopore Technologies, Oxford, UK).

### 2.5. Whole Genome Assembly and Analysis

ONT reads were assembled using Trycycler (accessed on 13 December 2022) [30]. The merged assembly consensus sequences were used for an initial taxonomy assignment against the bacterial genomes available from the NCBI RefSeq complete genomes database (22 February 2022) using Kraken2 [31]. The consensus sequences were also used to find the most closely related bacteria using the GTDB database (Release 214, version 2.3.0, May 2024). All closely related bacterial strains were used for an Average Nucleotide Identity (ANI) analysis utilising the pheatmap package in R [32]. These strains included both type strains and non-type strains. Type strains were included for taxonomic identification, while the non-type strains increase the resolution of further analysis. The results from this analysis were used to create a heatmap in OriginPro 2020 (64-bit, version 9.7.0.188) to confirm the taxonomy assignment through group average clustering and Pearson correlation distance. Prokka was used to annotate bacterial genes within the consensus sequence [33]. After taxonomy assignment and gene annotation, the consensus sequences were imported into the software Geneious Prime (version 2024.0.2). The *nif* operon was manually examined to ensure completeness using Geneious Prime.

### 2.6. Plant Nitrogen Response Assay and DNA Extraction

Four different seed batches of *C. clandestinus* were used for this growth experiment. As multiple N-fixing bacteria harbouring seed batches (cv. Whittet from Anco Seeds (NF+_1) and cv. Whittet from Williams Group (NF+_2)) and N-fixing bacteria lacking seed batches (cv. Whittet from McKays Seeds (NF−_1) and cv. Acacia plateau from Williams Group (NF−_2)) were only available for *C. cladestinus*, these seed batches were selected for growth experiments allowing for direct comparisons to understand the effects of harbouring N-fixing bacteria within the seed microbiome. A total of 120 seeds per seed batch were washed in sterile water and sown in a potting mix consisting of coarse perlite, fine perlite, and coarse vermiculite (Exfoliators (Aust.) Pty. Ltd.) (Melbourne, Australia) in a 2:2:1 ratio. For each seed batch, a total of 30 seeds were sown over three pots, which were grown under four different nitrogen conditions, resulting in 12 pots for each seed batch. These nitrogen conditions included 0.1 mM, 1 mM, 10 mM, and 25 mM of potassium nitrate (KNO_3_). With the 0.1 mM and 1 mM nitrogen concentrations being considered as low nitrogen conditions, 10 mM being considered as the optimal nitrogen condition, and 25 mM being considered as a high nitrogen condition [34]. Each of these treatments was prepared in combination with a nutrient mixture (MgSO_4_, 0.48 g/L; KCl, 0.298 g/L; CaCl_2_, 0.55 g/L; KH_2_PO_4_, 0.408 g/L; Fe-EDTA, 34.4 mg/L; MnCl_2_, 1.26 mg/L; ZnSO_4_, 1.62 mg/L; CuSO_4_, 0.32 mg/L; H_2_BO_3_, 3.09 mg/L; Na_2_MoO_4_, 41.18 μg/L). The nutrient mixture with different levels of nitrogen was applied once a week (~167 mL per pot). After a full week of growth, 10 seedlings of similar shoot lengths were selected and retained from the total of 30 seeds that were planted, while all the other seedlings were removed. The selected seedlings were allowed to grow further for seven weeks, for a total of eight weeks of growth before harvesting. During harvest, root and shoot length and weight measurements were taken, and root and shoot samples were obtained in 1.2 mL collection tubes (QIAGEN cat# 9560) (Hilden, Germany) containing a single sterile stainless-steel bead (3 mm) for subsequent DNA extractions. These samples were stored at −80 °C until the DNA extraction. The DNA was extracted using a QIAGEN MagAttract 96 DNA Plant Core kit (cat# 67163) (Hilden, Germany) as per the manufacturer’s instructions. The DNA was used to prepare 16S amplicon libraries for Illumina MiSeq sequencing. Root and shoot measurements were analysed in Origin Pro 2020 (64-bit, version 9.7.0.188) through a one-way ANOVA test to establish the significance of differences between seed batches.

### 2.7. Illumina MiSeq 16S Amplicon Sequencing

Amplicon libraries were prepared following the 16S Metagenomic Sequencing Library Preparation guide from Illumina with minor modifications. The amplicon PCRs were conducted using primers 515F (Forward, 5′-GTGCCAGCMGCCGCGGTAA-3′) and 806R (Reverse, 5′-GGACTACNVGGGTWTCTAAT-3′), targeting the V4 region of the 16S rRNA gene [35]. Peptide nucleic acid (PNA) PCR blockers (50 μM) from PNA BIO Inc. (Thousand Oaks, CA, USA) were added to reduce amplification of 16S rRNA gene sequences derived from the plant organelle genomes [36,37]. The PCR was performed following the Illumina protocol, except for the 16S amplicon PCR, where an extra step of PNA clamping was included at 75 °C for 10 s. The index PCR was conducted using the Illumina Nextera XT indices to apply combinatorial dual index pairs for sample identification as per instructions in the 16S Metagenomic Sequencing Library Preparation guide. Libraries were quantified using an HSD 1000 ScreenTape on TapeStation (Agilent technology cat# 5067-5584) (Santa Clara, CA, USA) to ensure that amplification of the target had occurred. After each PCR, a cleanup was performed using Promega ProNex Size-Selective Purification system beads (Cat# NG2001) (Madison, WI, USA). The amplified samples were subsequently normalised using a SequalPrep Normalisation Plate (96) kit (Applied Biosystems, cat# A10510-01) (Waltham, MA, USA). Samples were pooled and sequenced following the Illumina MiSeq System Denature (Illumina, San Diego, CA, USA) and Dilute Libraries Guide.

### 2.8. Data Analysis of 16S Amplicon Sequencing

Sequencing data of the 16S amplicon was analysed through a QIIME 2 pipeline (version 2022.2). Raw reads were trimmed and merged using the plugin “PANDAseq” [38]. The merged reads were imported by creating a manifest as single-read FASTQ sequences. Remaining mitochondrial and chlorophyll reads were removed from the data [39], and the QIIME 2 plugin “DADA2” was used for denoising, assembly of a feature table, and filtering of reads. The QIIME 2 “feature classifier” plugin [40] was used for the taxonomic assignment of amplicon sequence variants (ASVs) from the SILVA SSU database 138 (released 16 December 2019). The 16S data were further analysed through the QIIME 2 plugin “phylogeny”, which was used to generate rooted and unrooted phylogenetic trees through MAFFT and FastTree 2 [41,42]. An ASV table was created through BIOM for further analysis. The analysis included preparing a 100% bar chart and a heatmap with dendrogram (Pearson correlation) showing the most abundant ASVs within the profiles of 14 seed batches. For these figures, the ASVs within each seed batch after combining the 16 replicates with an abundance of at least 10% were selected as abundant ASVs (aASVs) and visualised using OriginPro 2020. In addition, the entire ASV table was used to prepare principal coordinate analysis (PCoA) plots, which were generated using RStudio (version 2024.04.2) using the vegan R package [43]. These plots were prepared based on the Bray–Curtis distance matrix, utilising the package DESeq2 using the normalised reads, and the distance was statistically quantified through permutational multivariate analysis of variance (PERMANOVA) tests. The ASV table was also used to perform differential abundance analysis with the R package Analysis of Compositions of Microbiomes with Bias Correction 2 (ANCOM-BC2) [44], examining ASVs that show a monotonically increasing trend and a monotonically decreasing trend compared to the 0.1 mM nitrogen treatment. This analysis excludes any ASVs whose abundances did not significantly increase or decrease between all three nitrogen concentrations. The results from the differential abundance analysis were shown in a heatmap prepared with Excel.

## 3. Results

### 3.1. Isolation of Nitrogen Fixing Bacteria and PCR Confirmation

**Figure 1:**
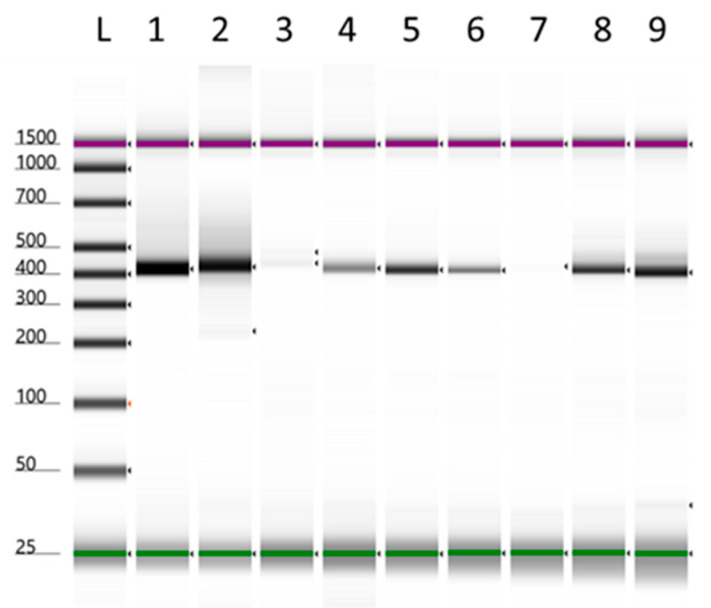
Agilent TapeStation HSD 1000 screentape results showing amplification of the *nif*H gene from three source cultures and six isolates. This figure includes a lower marker at 25 bp and an upper marker at 1500 bp. (1) Source culture of *Cenchrus clandestinus* (cv. Whittet, Anco Seeds). (2) Source culture of *C. clandestinus* (cv. Whittet, Williams Group). (3) Source culture of *Paspalum dilatatum* (Williams Group). (4) Isolate 1 from *C. clandestinus* (cv. Whittet, Anco Seeds). (5) Isolate 2 from *C. clandestinus* (cv. Whittet, Anco Seeds). (6) Isolate 1 from *C. clandestinus* (cv. Whittet, Williams Group). (7) Isolate 2 from *C. clandestinus* (cv. Whittet, Williams Group). (8) Isolate 1 from *P. dilatatum* (Williams Group). (9). Isolate 2 from *P. dilatatum* (Williams Group).

The isolation protocol implemented in this study included multiple growth stages, which has only resulted in growth after five days under the surface of the semi-solid media for three seed batches. The bacterial growth was plated out, and only the first dilution resulted in single colonies for three seed batches, with the remaining 11 seed batches not providing any bacterial growth. A total of six single colonies were picked based on their morphological appearance from three plates. These colonies came from seed batches, *C. clandestinus* (cv. Whittet, Anco Seeds), *C. clandestinus* (cv. Whittet, Williams Group), and *P. dilatatum* (Williams Group). These single colonies were used for colony PCR with primers amplifying the *nif*H gene. Alongside these single colonies, an aliquot of the source cultures was also included in the PCR. The samples, which initially showed amplification on a 2% agarose gel, were subsequently visualised using Agilent TapeStation (HSD 1000 ScreenTape) (Figure 1). Amplicons of the expected size (~400 bp) were observed, including clear amplification in the source culture sample of *C. clandestinus* (cv. Whittet, Anco Seeds) (lane 1), the source culture of *C. clandestinus* (cv. Whittet, Williams Group) (lane 2), the two isolates obtained from *C. clandestinus* (cv. Whittet, Anco Seeds) (lanes 4 and 5), an isolate obtained from *C. clandestinus* (cv. Whittet, Williams Group) (lane 6) and the two isolates obtained from *P. dilatatum* (Williams Group) (lanes 8 and 9). In addition, the source culture sample of *P. dilatatum* (Williams Group) produced amplification, but in very low concentration (lane 3). Similarly, the second isolate from *C. clandestinus* (cv. Whittet, Williams Group) produced a very faint band (lane 7). To sum up these results, six *nif*H-positive bacterial isolates from the warm-season grass seeds were found.

### 3.2. Taxonomic Assignment of Isolates

**Figure 2:**
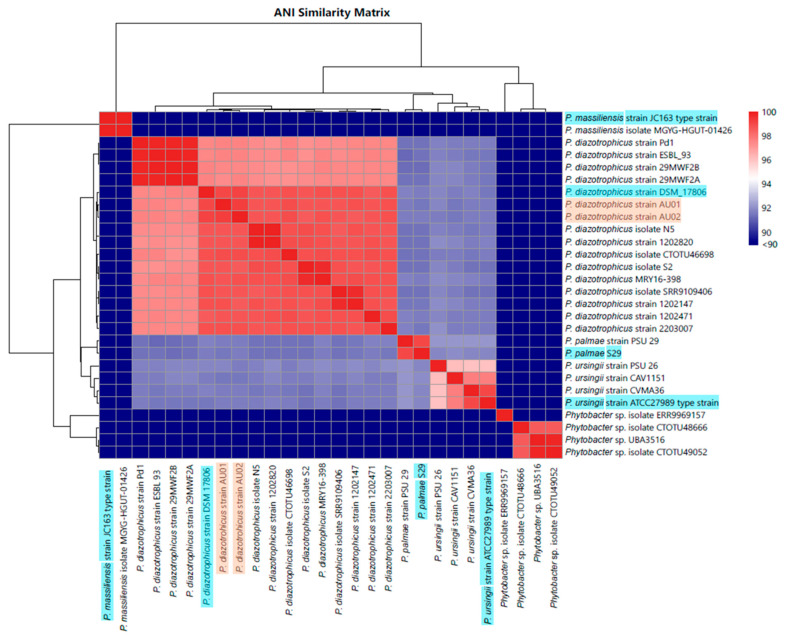
ANI comparison of the two isolated *P. diazotrophicus* strains. These two isolated strains were compared to both type strains and non-type strains based on the similarity to other *Phytobacter* species as generated by the GTDB database. Here, the isolates are shown in orange while the type strain of each species is shown in blue.

**Figure 3:**
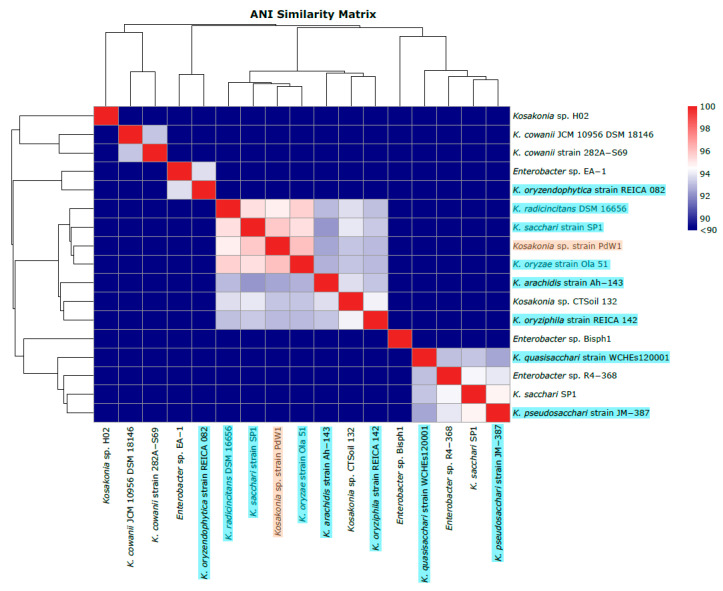
ANI comparison of the isolated *Kosakonia* sp. strain PdW1. This isolate was compared to both type strains and non-type strains based on the similarity to 13 other *Kosakonia* strains and three *Enterobacter* strains as generated by the GTDB database. Here, the isolate is shown in orange while the type strain of each species is shown in blue.

The six isolates that produced amplicons of the expected size were used for whole-genome sequencing, taxonomy assignment, and gene annotation. The whole genome sequencing results demonstrated that the two independently isolated cultures from each seed batch were genomically identical, leading to three different microbes (one per seed batch). Based on the Kraken2 results, the isolate from the seed batch *C. clandestinus* (cv. Whittet, Anco Seeds) was taxonomically most similar to *Phytobacter diazotrophicus* (strain AU01) (International Depositary Authority (IDA) accession V24/010671), as was the bacteria isolated from the seed batch *C. clandestinus* (cv. Whittet, Williams Group) (strain AU02) (IDA accession V24/010672). The bacteria isolated from the seed batch *P. dilatatum* (Williams Group) were taxonomically most similar to *Kosakonia* sp. (strain PdW1) (IDA accession V24/010673). The GTDB database was used as a tool to find the most closely related bacterial species to the three isolated bacteria. The GTDB database analysis indicated that there were 26 *Phytobacter* species (four type strains for each of the included species and 22 non-type strains), which were closely related to the two isolated *P. diazotrophicus* strains. For the isolated *Kosakonia* sp. strain PdW1, the GTDB database generated 13 *Kosakonia* species (eight type strains and five non-type strains) and three *Enterobacter* species (zero type strains and three non-type strains), which were closely related. These closely related strains were visualised in heatmaps with dendrograms based on ANI analysis results and were created within Origin Pro. The analysis, including the genus *Phytobacter*, showed that there was a total of five clades with a similarity of 95% and above (Figure 2). The clade containing the two isolated *P. diazotrophicus* strains included a range of different *P. diazotrophicus* strains, including the type strain (*P. diazotrophicus* strain DSM 17806). The percentage similarity of the two isolates compared to the type strain was 99.03% and 99.13% for strain AU01 and AU02, respectively. This indicates that these two bacteria were newly isolated strains of the species *P. diazotrophicus*. This is strengthened by the fact that there is a separate clade of *P. diazotrophicus*, sharing similarity of around 99%, which does not include a type strain, indicating this clade might be of a different species. The ANI analysis, including the genus *Kosakonia*, showed that there was only one clade with a similarity of between 95% and 96% (Figure 3). This clade contained the isolated *Kosakonia* sp. strain PdW1, as well as the type strains representing three other *Kosakonia* species, including *K. radicincitans*, *K. sacchari*, and *K. oryzae*, sharing a similarity of 94.93%, 95.84%, and 96.06% with *Kosakonia* sp. strain PdW1, respectively. Such results indicate that the *Kosakonia* isolate does not fall into a clade with a specific species, and hence the isolate was designated *Kosakonia* sp. This is also strengthened by the fact that there are more type strains included in this analysis, as the species of the isolated *Kosakonia* is not yet well defined at this stage. All type strains included in this analysis were confirmed through utilising the TYGS tool (https://tygs.dsmz.de/ (accessed on 17 February 2026)). The results of this analysis are included in the Supplementary Materials. Here, the results are shown as digital DNA:DNA hybridization (dDDH) values based on Genome-to-Genome Distance Calculator (GGDC) supporting the assigned taxonomies for all three isolates [45].

### 3.3. Gene Annotation of Isolates

**Figure 4:**
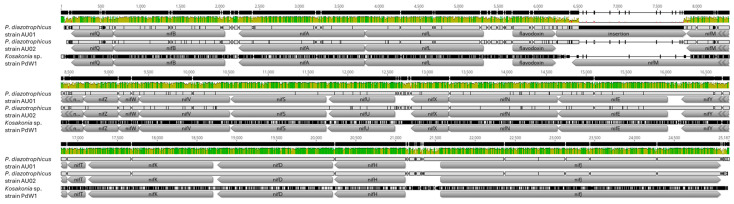
The aligned nif operons of the three isolates contain all genes that are vital for nitrogen fixation. *P. diazotrophicus* strain AU01 contains an insertion that is not present within *P. diazotrophicus* strain AU02 or *Kosakonia* sp. strain PdW1. The alignment shows pairwise identities of 94.1% between the two *P. diazotrophicus* strains, 73.6% between *P. diazotrophicus* strain AU01 and *Kosakonia* sp. strain PdW1, and 77.6% between *P. diazotrophicus* strain AU02 and *Kosakonia* sp. strain PdW1.

*P. diazotrophicus* strain AU01 produced a circular genome containing 5,586,165 bp (GC content, 53.1%) with 5319 annotated genes. For *P. diazotrophicus* strain AU02, the genome included a chromosome with a length of 5,590,470 bp (GC content, 53.0%) and a plasmid with a length of 41,010 bp (GC content, 47.8%). These two DNA molecules contained a total of 5372 annotated genes. The *Kosakonia* sp. strain PdW1 genome contained two circular segments as well, with the chromosome being 5,502,910 bp (GC content, 54.3%) and the plasmid being 132,415 bp (GC content, 52.2%), containing a total of 5338 annotated genes. The sequences were imported into Geneious Prime, and the annotated *nif* operon was examined for all three isolates. All essential *nif* genes (*nif*H/D/K/E/N/B) for these bacteria to fix atmospheric nitrogen were present in all three isolates (Figure 4) [46]. In addition, each of the three isolates contains the genes *nif*Q, A, L, M, Z, W, V, S, U, X, Y, T, J, and F (Flavodoxin). This indicates that these isolates should be able to fix atmospheric nitrogen around the roots of warm-season pasture grasses. While the three isolates contain the same set of *nif* genes, these operons were not genetically identical. *P. diazotrophicus* strain AU01 contains an insertion of 1692 bp between 2,681,171 and 2,682,799 bp located within the *nif* operon. This insertion was the main difference between *P. diazotrophicus* strain AU01 and strain AU02, containing a pairwise identity of 94.1% between the two *nif* operons. The *nif* operon present in *Kosakonia* sp. strain PdW1 was quite different from *P. diazotrophicus* strain AU01 and strain AU02, with pairwise identities of 73.6% and 77.6%, respectively. The genomes of the isolates are publicly available on NCBI GenBank under BioProject PRJNA1397244.

### 3.4. C. clandestinus Pot Assay

**Figure 5:**
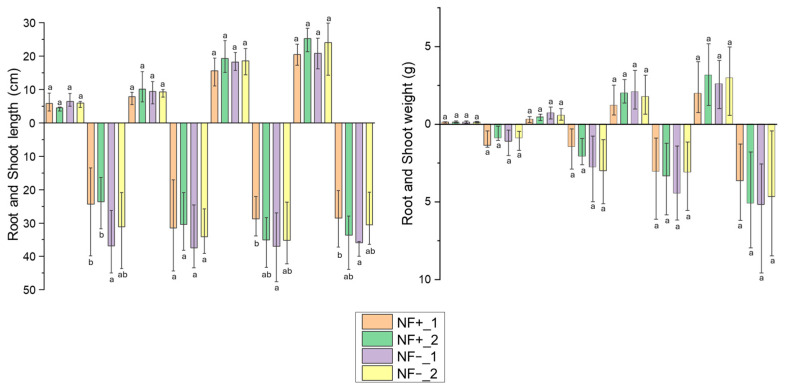
Freshly harvested plant length and weight measurements were taken from roots (measurements shown below 0-axes) and shoots (measurements shown above 0-axes) of the four different *C. clandestinus* seed batches. These seed batches include *C. clandestinus* cv. Whittet from the distributor McKays Seeds (NF+_1), cv. Whittet from the distributor Anko seeds (NF+_2), cv. Whittet from the distributor Williams Group (NF−_1) and cv. Acacia Plateau from the distributor Williams Group (NF−_2). The figure includes significant differences indicated by different letters (a and b). The measurements show significant root length differences between NF+_1 and NF+_2 compared to NF−_1 when grown under 0.1 mM nitrogen conditions. There was a significant root length difference between NF+_1 and NF−_1 when grown under 10 mM and 25 mM nitrogen conditions.

A pot assay was performed under glasshouse conditions to examine whether there were different responses to nitrogen between *C. clandestinus* seed batches from which potential N-fixing bacteria had been isolated (NF+_1, NF+_2), and those from which no N-fixing bacteria could be isolated (NF−_1, NF−_2). 10 seeds from each batch were grown under four different nitrogen concentrations. After eight weeks of growth, the freshly harvested *C. clandestinus* plants from the four different seed batches were immediately measured for their fresh shoot (shown above 0-axes) and root (shown below 0-axes) length and weight. The measurements, seen in Figure 5, show clear differences between the shoot lengths and weights of the plants that were grown under the different nitrogen conditions. For roots, the length did not show a clear trend of increasing when the N concentration increased, but this trend was clear with the root weights. Comparing between seed batches within each nitrogen condition, the roots of the seed batch *C. clandestinus* cv. Whittet from the distributor McKays Seeds (NF−_1) were significantly longer when compared to the roots from the NF+ batches under the low nitrogen concentration of 0.1 mM (*p* < 0.05), with the NF−_2 also being longer but not statistically so. While under higher nitrogen treatments (10 mM and 25 mM), a significant difference in root lengths was still seen between NF−_1 and NF+_1. None of the other comparisons showed any significant differences between them.

### 3.5. Illumina MiSeq 16S Amplicon Sequencing Analysis

#### 3.5.1. Bacterial Community Comparison—Beta Diversity Analysis

**Figure 6:**
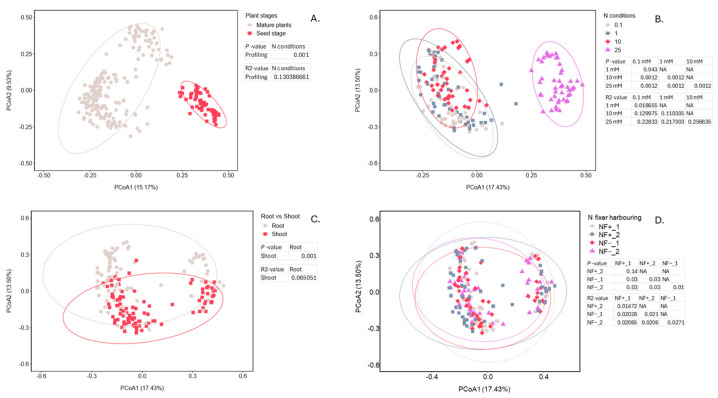
PCoA plot of bacterial communities, including the PERMANOVA results showing the *p*-value indicating the significance of community differences and the R^2^-value representing the degree of separation between all parameters shown in the figures. (**A**) PCoA plot comparing the seed bacterial microbiome with the bacterial communities present in mature plants under different nitrogen conditions. (**B**) PCoA plot comparing the microbial communities present under four different nitrogen conditions. (**C**) PCoA plot comparing the microbial communities present in the roots and shoots of the plant samples. (**D**) PCoA plot comparing the microbial communities of four different *C. clandestinus* seed batches with two seed batches harbouring potential N-fixing bacteria and two seed batches lacking N-fixing bacteria. The ellipse represented 95% confidence interval.

The drivers of microbial communities were assessed through PERMANOVA and PCoA. This analysis was done to compare microbial communities present in the initial seed microbiomes [26] and within the roots and shoots from the experiment examining the seed batches grown under four different nitrogen conditions (Figure 6). Significant differences in microbial communities were represented by a *p*-value < 0.05, and the degree of separation between communities was represented by R^2^-values. Comparisons to the seed microbiome show that the microbial communities undergo a significant change (*p* = 0.001) from the initial microbiomes found in the seeds to the microbiomes of the roots and shoots of plants grown under different nitrogen conditions (Figure 6A). Figure 6B shows the separation based on the four nitrogen conditions, with significant differences between all four treatments being detected (*p* < 0.05). The R^2^-values confirm that the 25 mM treatment was more different from the other three N treatments (R^2^ > 0.2). For the other three treatments, the R^2^-values were much lower, indicating less of a difference between these treatments (R^2^ < 0.2). The root and shoot communities (Figure 6C) were significantly different (*p* = 0.001). The community comparison between the plants grown from the four different seed batches (Figure 6D) under different N conditions shows no significant difference between those from NF+_1 and NF+_2 plants (*p* = 0.139), while there were significant differences (*p* < 0.05) between the other comparisons. This demonstrates that the bacterial communities of the potential N-fixing bacteria harbouring seed batches were more similar than those of seed batches that did not harbour any N-fixing bacteria.

#### 3.5.2. Bacterial Community Changes—Differential Abundance Analysis

**Figure 7:**
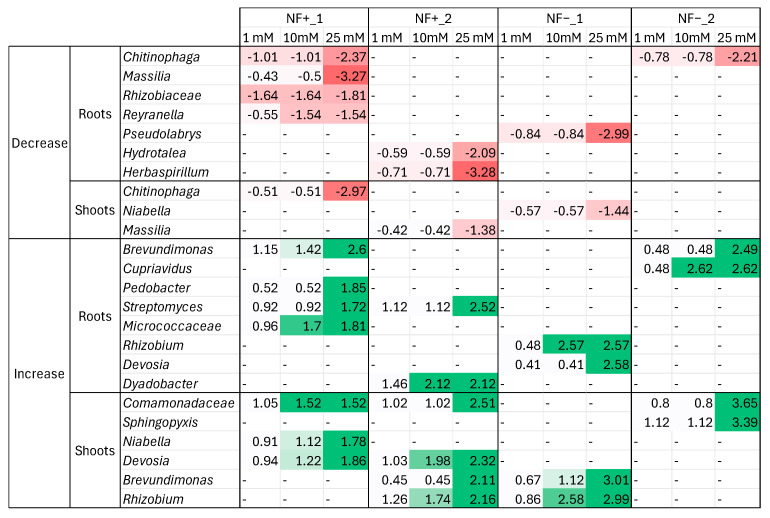
Differential abundance analysis shows monotonically increasing (in green) and decreasing (in red) ASVs from 0.1 mM compared to the other three higher nitrogen conditions on the genus level within all four seed batches for the roots and shoots.

**Figure 8:**
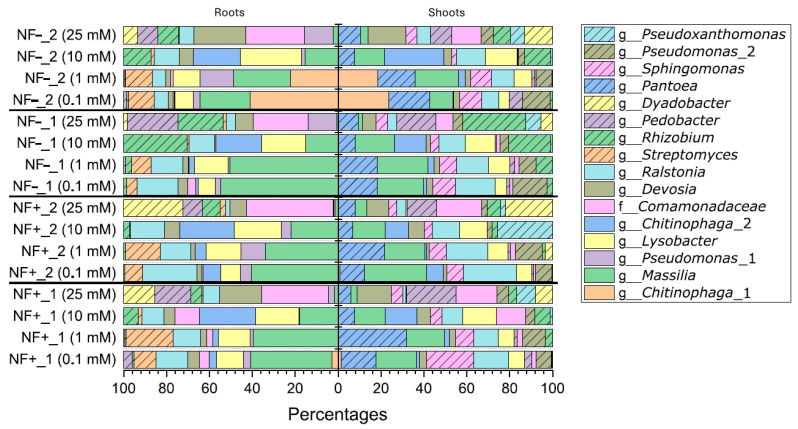
100% bar chart containing the 10% most abundant ASVs present in the shoots and roots of the four *C. clandestinus* seed batches grown under all four nitrogen concentrations. This figure shows the bacterial profiles of *C. clandestinus* cv. Whittet from Anco Seeds (NF+_1), *C. clandestinus* cv. Whittet from the Williams Group (NF+_2), *C. clandestinus* cv. Whittet from McKays Seeds (NF−_1) and *C. clandestinus* cv. Acacia plateau from the Williams Group (NF−_2) grown under four different nitrogen treatments.

A differential abundance analysis of microbiome profiles was performed to uncover ASVs that significantly changed in abundance when seeds were grown under four different nitrogen conditions. ASV abundances at 1 mM, 10 mM, and 25 mM were compared to those at 0.1 mM treatment levels (Figure 7), and only ASVs whose abundances had monotonic increasing or decreasing trends when N concentration increased were shown. In this analysis, the ASVs from potential N-fixing bacteria harbouring seed batches (NF+_1 and NF+_2) and no N-fixing bacteria harbouring seed batches (NF−_1 and NF−_2) for both roots and shoots were considered. Figure 7 shows that NF+_1 and NF+_2 contain more monotonic increasing and decreasing trending ASVs compared to NF−_1 and NF−_2. Seed batches NF+_1 and NF+_2 contain five and three monotonically decreasing ASVs and seven and five monotonically increasing ASVs, respectively. NF−_1 and NF−_2 only contain two and one monotonically decreasing ASVs, respectively, and four monotonically increasing ASVs each. The fact that there are more monotonic increasing trends visible compared to monotonic decreasing trends indicates there are fewer different ASVs utilised by the plants under low nitrogen conditions, focusing more on recruiting particular microbes to aid plant growth under these harsh conditions. Some of these ASVs are assigned to the genera *Chitinophaga*, *Massilia*, and *Herbaspirillum*, which have all been shown to improve plant growth under low nitrogen conditions [47,48,49]. In contrast, genera like *Brevundimonas* and *Pedobacter*, which increase in abundance under the higher N-conditions, have been reported to improve plant growth under drought and more favourable conditions [50,51]. The ASVs from the assigned genera *Chitinophaga*, *Massilia*, and *Rhizobium* and family *Comamonadaceae* were also highly abundant and found within the abundant ASV profiles (Figure 8).

#### 3.5.3. Bacterial Profiles—Most Abundant ASVs

The microbial profiles of the dominant ASVs are shown in a 100% bar chart, which contains the most abundant ASVs (aASVs, abundance higher than 10% over the six replicates for each seed batch under each of the nitrogen conditions within the roots and shoots; Figure 8). The aASVs present in the seed batches were taxonomically assigned at the genus level and, in some cases, the family level. Within the profiles, there were a total of 16 aASVs present, which were distributed over the roots and shoots. Two aASVs, which were assigned to the genera *Massilia* and *Lysobacter,* were reduced in abundance with increasing nitrogen concentrations and disappeared under the 25 mM nitrogen concentration within both roots and shoots. A similar trend can be seen for the aASV of the genus *Ralstonia,* which decreases with increasing nitrogen levels, but in contrast to the *Massilia* and *Lysobacter* ASVs, it was still present at low abundance at the 25 mM nitrogen condition for both roots and shoots. These aASVs, which were mostly outcompeted at the 25 mM nitrogen condition in both roots and shoots, were replaced by a higher abundance of aASVs from the family *Camomonadaceae* and the genera *Devosia*, *Rhizobium*, *Dyadobacter,* and *Pedobacter*. An aASV from the genus *Chitinophaga* (*Chitinophaga*_1) was only present in NF-_2 grown under 0.1 mM and 1 mM nitrogen conditions for both roots and shoots. The genera *Pantoea*, *Sphingomonas*, *Pseudomonas* (*Pseudomonas*_2), and *Pseudoxanthomonas* were restricted to the shoots of all the seed batches and present in all nitrogen conditions, except for *Pseudoxanthomonas,* which was only present in the 25 mM nitrogen conditions. A *Streptomyces* ASV was restricted to the roots of all four different seed batches when grown under 0.1 mM and 1 mM nitrogen conditions.

#### 3.5.4. Presence of Isolated N-Fixing Bacteria

**Figure 9:**
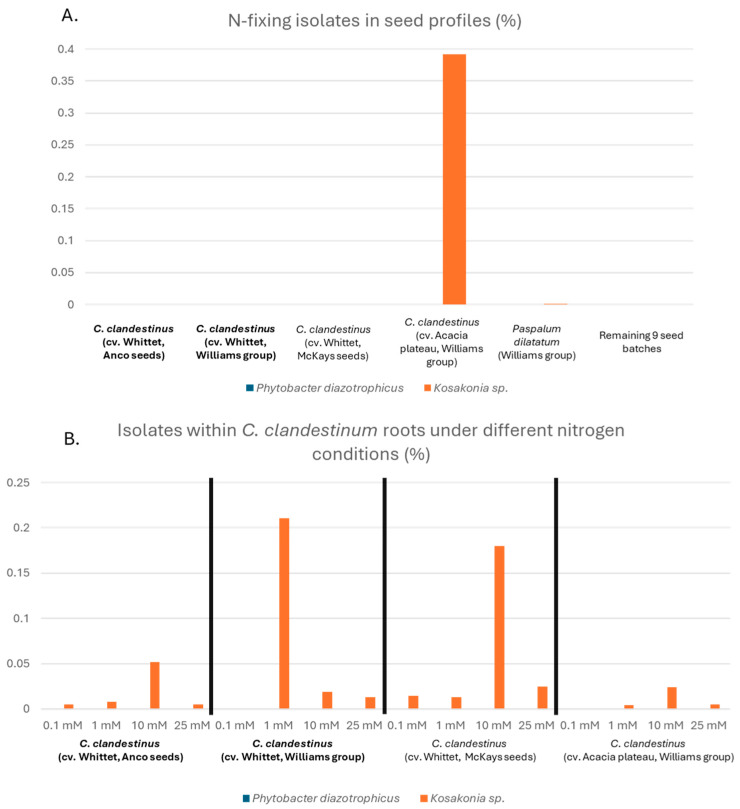
Bar chart showing the presence of ASVs containing an identical 16S rRNA V4 region as the isolated *P. diazotrophicus* strains and *Kosakonia* sp. strain PdW1 within different seed batches. (**A**) Presence of the ASVs with the same 16S V4 region as the isolates within the seed microbiome of all 14 seed batches, including the two potential N-fixing bacteria harbouring seed batches, NF+_1 and NF+_2 (shown in bold font), and the two seed batches lacking N-fixing bacteria NF−_1 and NF−_2. (**B**) Presence of ASVs with identical 16S rRNA V4 regions as the isolates within the root microbiome of the four *C. clandestinus* seed batches grown under four different nitrogen conditions, including the two seed batches harbouring N-fixing bacteria, NF+_1 and NF+_2 (shown in bold font), and two seed batches which did not harbour any N-fixing bacteria, NF−_1 and NF−_2.

The abundance of ASVs representing the isolated potential N-fixing bacteria within the seed microbiome and within *C. clandestinus* grown under different nitrogen conditions is visualised in bar charts (Figure 9). Figure 9A shows the presence of these ASVs within the seed microbiome described in our previous study [26]. This figure shows that there were no ASVs present within the microbiomes of 16 seed replicates identical to the 16S rRNA V4 region of the two *P. diazotrophicus* strains within the 14 seed batches. An ASV with an identical 16S rRNA V4 to the isolated *Kosakonia* sp. strain PdW1 was present in two seed batches. These seed batches include *C. clandestinus* cv. Acacia plateau from Williams Group and *Paspalum dilatatum* from Williams Group (host batch of the isolated *Kosakonia* sp.), with 367 reads (0.39% of total reads) and 16 reads (0.001% of total reads), respectively. Similarly, no ASV with an identical 16S rRNA V4 region to the *Phytobacter* isolates was found in the root samples of the four *C. clandestinus* seed batches growing under different nitrogen conditions (Figure 9B). An ASV with an identical 16S rRNA V4 region to the isolated *Kosakonia* sp. strain PdW1 was present at different nitrogen conditions within all four seed batches, despite no N-fixing *Kosakonia* being isolated from these seed batches. While this ASV was present in all four seed batches, its abundance under different N levels of each seed batch varied between 0% (0 reads) and 0.22% (183 reads). In addition, there is no evidence of a strong N response pattern. Interestingly, the seed batch *C. clandestinus* cv. The Acacia plateau from the Williams Group had the highest number of *Kosakonia* ASV reads in the seed microbiome profile, while containing the lowest number of reads after eight weeks of growth.

## 4. Discussion

### 4.1. Potential Nitrogen Fixing Bacterial Isolations

The isolation method used in this study is based on multiple isolation stages to select for growth of specific bacterial strains that are able to grow in low-N, low O_2_ conditions [51]. This isolation method has previously been found to be successful in isolating N-fixing members of the genera *Paenibacillus* and *Herbaspirilum* [27,52]. The initial isolation stage is based on the growth of ground seeds within liquid N-free media. During this stage, the bacteria are expected to grow based on the nutrient availability from the seeds present in the media, and it is not considered the selective stage of the isolation method [52]. The semi-solid agar stage allows N-fixing bacteria to grow away from the surface, enabled by a low O_2_ level suitable for N-fixing bacterial growth. This is due to the fact that diazotrophic bacteria and their ability to fix nitrogen can be affected by O_2_ through oxygen-mediated denaturation of the nitrogenase complex [53]. During the semi-solid selection stage, diazotrophic bacteria initially grow towards the middle of the media where there is little oxygen available and grow towards the surface when the level of bacteria increases to support aerobic respiration [54]. Employing this methodology has confirmed the presence of potential diazotrophic bacteria within the seed microbiome of some warm-season grasses, similar to the cool-season pasture grass, perennial ryegrass [27]. Both our study and the study performed by Li et al. (2021) [27] required the use of selective media to enrich for N-fixing bacteria, indicating these bacteria are difficult to isolate. In both cases, 1000 seeds were used, indicating that these bacteria are not only difficult to isolate but are also rare members of the seed microbiome. This is also supported by the fact that the two isolated *P. diazotrophicus* strains were absent within 16 seed replicates used for each seed batch discussed in van Essen et al. (2025) [26]. Previous experimentation has also shown that even though bacterial isolates could be considered as classical nitrogen-fixing bacteria does not always imply that these bacteria can actually fix atmospheric nitrogen with certain strains within a species lacking an effective nitrogenase complex [54]. One limitation of the isolation method employed in this study was that only sucrose was used as a carbon source, which has previously been found to mainly support fast-growing isolates [55]. Different carbon sources and longer bacterial growth development could enhance the growth of slower-growing isolates and would need to be investigated further in future research to assess if there might be any other potential N-fixing bacteria present within the seed batches.

### 4.2. Isolated Nitrogen Fixing Bacteria

The bacteria isolated in this study all contained the core genes for these bacteria to perform nitrogen fixation [46]. However, the genomic analysis revealed differences between the three isolates and the effect of these differences between the *nif* operons on the ability of these bacteria to fix nitrogen and their subsequent effect on plant growth will need to be investigated further [56,57]. *P. diazotrophicus* has been described in a previous study, isolated from the rhizosphere of date palms in the United Arab Emirates [58]. This isolate also contains a *nif* operon similar to the isolates described in our study. This isolate, however, similarly to our isolates not yet been investigated further to confirm its plant growth-promoting traits. The genus *Kosakonia* has previously been isolated from the warm-season pasture grass *Cenchrus purpureus* (Napier grass) and characterised as diazotrophic bacteria [59]. This genus has been studied in more depth and has previously been found to contain multiple plant growth-promoting traits [60]. Within these traits, atmospheric nitrogen fixation is well understood, which indicates the possibility of applying *Kosakonia* sp. strain PdW1 as a biofertiliser. However, the fact that two *P. diazotrophicus* strains were independently isolated from the same cultivar of *C. clandestinus* shows these microbes have a possible role in plant growth promotion for this grass. Previous research [61] demonstrated that the presence of native diazotrophic bacteria enhances plant growth under low nitrogen conditions. Understanding the ability of *C. clandestinus* seed batches harbouring the *P. diazotrophicus* strains to grow under nitrogen-deficient conditions compared to the *C. clandestinus* seed batches lacking N-fixing bacteria could give insights into the role of the *P. diazotrophicus* strains in *C. clandestinus* growth. However, further research needs to be performed to understand the ability of these bacterial isolates to fix atmospheric nitrogen and promote plant growth, as at this stage, there is no proof of N-fixation effectiveness and gene expression of the essential *nif* genes under nitrogen-deficient conditions.

### 4.3. Ability of C4 Grasses to Grow Under Low Nitrogen Conditions

The statistically shorter root lengths of the two seed batches harbouring potential N-fixing bacteria (NF+_1 and NF+_2) when grown under low nitrogen conditions could be a result of NF+_1 and NF+_2 having a microbiome suitable for scavenging nitrogen. This could help plants focus their energy on growing shoots and maintain yield [62]. Noticeably, the short roots do not translate into a significant length and weight decrease within *C. clandestinus* shoots, as would be expected with limiting root growth. It has previously been found that plants harbouring diazotrophic bacteria can maintain consistent growth even with comparatively smaller root systems because the microbes supply nitrogen directly to the host, reducing the plants’ need to invest carbon into extensive root growth [63]. The contribution of the *C. clandestinus-associated* microbiome to the ability of these grasses to grow under limiting N conditions warrants further investigation, given that the two NF+ seed batches had similar microbiomes, as was shown in the PERMANOVA analysis. One possibility is that these batches were produced under higher levels of N stress, as has been shown for other stresses, e.g., drought, that the seeds recruit beneficial microbes at the seed production stage to cope with that stress [64]. The possibility of vertical transmission of the bacteria associated with the nitrogen cycle under different nitrogen conditions would need to be investigated further in a future study.

### 4.4. Presence of Isolated Nitrogen Fixing Bacteria

We were successful in isolating two *P. diazotrophicus* strains from two seed batches. However, ASVs with identical 16S rRNA V4 regions as the two *P. diazotrophicus* strains were not found in either the seed microbiomes described in van Essen et al. (2025) [26] or in the assay with the four *C. clandestinus* seed batches grown under different nitrogen conditions. Indicating, these microbes are very rare within the seed batches, and either present in only a small number of seeds at high abundance or present in low abundance within a larger number of seeds. Similarly, in a previous study on isolating N-fixing bacteria from perennial ryegrass, multiple bacteria with N-fixation capabilities were isolated, while these microbes were not found in the seed microbiome [27,65] until enrichment media were used. *P. diazotrophicus* has previously been isolated from young stems and nodal roots of wild rice (*Oryza rufipogon*) grown under field soil conditions [66]. It could be that the perlite and vermiculite growth media are not as conducive for colonisation by *P. diazotrophicus* compared to field soil. However, diazotrophic bacteria, including the genera *Azospirillum*, *Bacillus*, *Beijerinckia*, *Bradyrhizobium*, *Erwinia*, *Enterobacter*, *Herbaspirillum*, and *Gluconoacetobacter,* are found to colonise within the intercellular spaces and xylem vessels of plant roots in soil, showing that the soil mixture should not affect bacterial colonisation [9]. The fact that no ASV was found with the same 16S rRNA V4 region as the *P. diazotrophicus* strains indicates how rare these N-fixing isolates are within the seed batches. An ASV with an identical 16S rRNA V4 region as the isolated *Kosakonia* sp. strain PdW1 was found to be present both in the seed microbiome and in the initial assay with four *C. clandestinus* seed batches. This ASV was most abundant in the seed microbiome within *C. clandestinus* cv. Acacia plateau from Williams Group, while also present in most of the *C. clandestinus* seed batches when grown under different nitrogen conditions. *Kosakonia* sp. strain PdW1 was isolated from the seed batch *Paspalum dilatatum*, not from the *C. clandestinus* seed batches, indicating there might be an ASV present within *C. clandestinus* seed batches with an identical 16S rRNA V4 region to the isolate, which could be lacking a *nif* operon needed to fix atmospheric nitrogen, as we were not able to isolate this microbe. *Kosakonia* is a very common genus with multiple similar species, which are hard to distinguish based only on the V4 region of the 16S rRNA gene [67]. Further research on applying these microbes to seeds in higher concentrations to improve their ability to colonise the root systems of warm-season pasture grasses or other economically important crops would be of great interest to understand the possibility of utilising these microbes as biofertilisers in the future.

### 4.5. Bacterial Communities Present Under Different Nitrogen Conditions

Even though the *P. diazotrophicus* strains were not present within *C. clandestinus* seed batches grown under different nitrogen conditions, bacterial communities are known to be highly adaptable based on the environmental conditions [68,69,70]. An example is the enrichment of diazotrophic bacteria around warm-season pasture grass roots only when grown under nitrogen-deficient soil conditions [23]. When comparing the microbial communities present within the plants grown under the four different nitrogen conditions, our results show that plants grown under the lower nitrogen conditions contain significantly different microbial communities compared to the plants grown under the highest nitrogen condition. The ASVs, which were highly abundant within the plants grown under the low nitrogen conditions, came from the genera *Chitinophaga*, *Massilia*, *Lysobacter*, *Streptomyces*, *Pantoea*, and *Sphingomonas*. These genera, except for the genus *Sphingomonas*, have all been described to be involved in the nitrogen cycle, either through N acquisition (*Chitinophaga*, *Massilia*, *Streptomyces*, and *Pantoea*) or (de)nitrification (*Lysobacter*) [47,48,71,72,73]. While the genus *Sphingomonas* has not been reported to be involved in the nitrogen cycle, it has been shown to improve plant growth under drought conditions [74,75]. This indicates that the microbial communities were selected based on the environmental conditions, with many of these microbes being known to improve nitrogen levels in the soil. For instance, the genus *Chitinophaga,* which was present in higher abundance under lower nitrogen conditions, has been shown to be a chitinolytic microbe that is able to convert chitin into carbon and nitrogen [76,77]. It may be that chemical generation of N from chitin breakdown is preferred in the growth conditions used, compared to atmospheric nitrogen fixation. However, these particular microbes might be interacting with each other and with the plant host and form a specific community [78]. Understanding the balance between differing methods of acquiring N from the environment within plant microbial communities will need to be investigated further in future work. The plants grown under higher nitrogen concentration contain the ASVs belonging to the genera *Devosia*, *Pedobacter*, *Rhizobium*, and *Dyadobacter*, in high abundance. These particular ASVs are known to possess a large range of properties that aid plant health under a range of different conditions, including plants grown under nitrogen-deficient conditions [50,79,80,81,82]. The predominant microbes within the root and shoot communities of the warm-season pasture grasses, under varying nitrogen conditions, consist of growth-promoting microorganisms. Understanding the host-microbe interactions under both low and high nitrogen conditions could be vital to address potential gaps to aid plant growth and health. In addition, from these abundant ASVs under the different nitrogen conditions, the ASVs from the assigned genera *Chitinophaga*, *Massilia*, and *Rhizobium* and family *Comamonadaceae* had significant changes in abundance within these profiles, as will be discussed in more detail within the differential abundance analysis section.

### 4.6. Changes in Bacterial Communities When Grown Under Different Nitrogen Conditions

A previous study has shown that the seed microbiome is maintained in the rhizosphere of a range of juvenile crops when grown in sterile sand [83]. However, in this study, the plants grew under sterile and favourable conditions, excluding environmental bacteria and changes in the microbiome induced by stress. In contrast, other studies have shown that under field conditions, the rice seed microbiome is preserved, but there is a large increase in environmental microbes that can be found around the rhizosphere of rice plants [84,85]. In our previous study, we identified 19 highly abundant ASVs on the genus level within the seed microbiome profiles from the *C. clandestinus* seed batches [26]. From these 19 ASVs, only three were present in high abundance within the root profiles of the same four seed batches grown under different nitrogen conditions. From the three abundant ASVs, two were from the assigned genus *Chitinophaga* and one was from the assigned genus *Rhizobium*. One of these *Chitinophaga* ASVs (*Chitinophaga*_1 from Figure 8) was highly abundant at the 0.1 mM and 1 mM treatment of *C. clandestinus* cv. Acacia plateau from Williams Group. The second *Chitinophaga* ASV (*Chitinophaga*_2 from Figure 8) was highly abundant at the 10 mM treatment for all four seed batches. The *Rhizobium* ASV was highly abundant at the 10 mM and 25 mM treatments of *C. clandestinus* cv. Acacia plateau from Williams Group and cv. Whittet from McKays Seeds. Both *Chitinophaga* and *Rhizobium* species are known to possess beneficial effects on plants under nitrogen-deficient conditions [47,50,81]. Our study showed that the majority of the *C. clandestinus* seed microbiomes were outcompeted by environmental microbes. Changes in seed-associated microbiota have also been studied in *Astragalus mongholicus*, where the seed microbiome changes under stress [86]. The significant impact of the environment on these bacterial communities was also supported by the comparisons of communities between the seed microbiome and when growing the same seed batches under different nitrogen conditions.

### 4.7. Differential Abundance Analysis of Bacterial Communities Within C. clandestinus

The ASVs enriched within NF+_1 and NF+_2 at higher nitrogen conditions were from the assigned genera *Brevundimonas*, *Pedobacter*, *Streptomyces*, *Dyadobacter*, *Niabella*, *Devosia*, and *Rhizobium*, and the assigned families *Micrococcaceae* and *Comamonadaceae* when grown under higher nitrogen conditions compared to the lower nitrogen conditions. The ASVs from the assigned genus *Rhizobium* and family *Comamonadaceae* are present in high abundance within the profiles as well. While the ASVs that were present in lower abundance at the higher nitrogen conditions compared to the lower nitrogen conditions included the assigned genera, *Chitinophaga*, *Massilia*, *Reyranella*, *Hydrotalea*, and *Herbaspirillum*, and the assigned family *Rhizobiaceae*. With the ASVs from the assigned genera *Chitinophaga* and *Massilia* also being present in high abundance within the profiles. From ASVs whose abundance increased, the genus *Brevundimonas* is a microbe that has been shown to enhance plant growth under arid conditions [51], while the genus *Pedobacter* has been shown to have plant growth-promoting properties under “good” soil conditions [50]. In a study on canola and soybean, two bio-stimulant compounds extracted from a *Devosia* sp. showed the ability to enhance plant growth under optimal conditions and under saline conditions [87]. This could explain the enrichment of the abundance of *Brevundimonas*, *Pedobacter*, and *Devosia* under higher nitrogen conditions, as nitrogen availability is not an issue; microbes are enriched, which supports plant growth under less significant deficiencies. As for the ASVs whose abundance decreased under the high nitrogen conditions, the genus *Chitinophaga* was previously discussed as being involved in N-acquisition, while the genus *Massilia* has been shown to be involved in the flowering and overall biomass of maize [88]. The plants under low nitrogen conditions were flowering relatively early due to the stress, which could explain why the ASV of the genus *Massilia* was enriched under these low nitrogen conditions. The genus *Herbaspirillum* contains species that are found to be diazotrophic bacteria, hence their enrichment under low nitrogen conditions [49]. In addition, it is found that certain *Herbaspirillum* sp. in low abundance also drive microbial changes around plants to improve plant growth [89]. This could be similar to what happened with the *P. diazotrophicus* strains, which were present in low abundance as diazotrophic bacteria but could be driving the changes in microbial communities. This would need to be investigated further to see the effect of these isolates in possibly driving microbial changes to benefit plants under different conditions.

## 5. Conclusions

This paper describes the isolation of three different bacteria with *nif* operons containing all essential genes for nitrogen fixation from warm-season pasture grass seeds. As seen in the cool-season pasture grass, perennial ryegrass, these microbes were rare in the seed microbiome and needed to be selectively isolated. There were two hypotheses about their rarity: they could either be present below the level of detection in many seeds within a batch, or they could be present at reasonable abundance levels in a few seeds. The inability to detect these microbes in plants grown from seed batches that harbour potential N-fixing bacteria grown under low nitrogen conditions, which should be favourable to N-fixers, points to them being present in a few seeds. Similar root phenotype and bacterial microbiome population responses under limiting nitrogen of seed batches harbouring potential N-fixers may point to the rare bacteria being an indicator of a more nitrogen-responsive microbiome. The role of the potential N-fixers, along with other members of the nitrogen-responsive microbiomes, needs to be investigated further for the development of biological fertilisers utilising these bacteria. To date, *Kosakonia* species have already been used as biofertilisers, indicating the potential of these bacteria [90].

## Figures and Tables

**Table 1:** List of warm-season grass seed batches used in this study, including species name, common name, distributors, cultivars, assigned seed batch names, and geographical location of seed production [26].

Species	Common Name	Distributor	Cultivar	Seed Batch Name	Geographical Location
*Cenchrus clandestinus* (Hochst. ex Chiov.) Morrone	Kikuyu grass	McKays Seeds Anco Seeds Williams Group	Whittet Whittet Whittet Acacia Plateau	CcWM CcWA CcWW CcAW	North-eastern New South Wales, Australia North-eastern New South Wales, Australia North-eastern New South Wales, Australia North-eastern New South Wales, Australia
*Chloris gayana* Kunth	Rhodes grass	McKays Seeds Barenbrug Williams Group	Katambora Katambora Mariner Tolgar Endura Katambora Callide Excel	CgKM CgKB CgMB CgTB CgEB CgKW CgCW CgEW	South-eastern Queensland, Australia Northern Queensland, Australia Northern Queensland, Australia Northern Queensland, Australia Northern Queensland, Australia South-eastern Queensland, Australia North-eastern New South Wales, Australia North-eastern New South Wales, Australia
*Paspalum dilatatum* Poir.	Dallis grass	Williams Group		PdW	Arizona, USA
*Paspalum notatum* Flüggé	Bahia grass	McKays Seeds	Pensacola	PnM	Eastern New South Wales, Australia

## Data Availability

The original data presented in the study are openly available in the NCBI GenBank under the BioProject PRJNA1397244. In addition, the isolated strains have been deposited with the International Depository Authority under the ‘Budapest Treaty on the International Recognition of the Deposit of Microorganisms for the Purposes of Patent Procedure’. The accession numbers assigned by the depository to the isolates were V24/010671, V24/010672, and V24/010673.

## Supplementary Materials

The following supporting information can be downloaded at: https://www.mdpi.com/article/10.3390/microorganisms14040786/s1, Supplementary File S1: FASTA file of *P. diazotrophicus* strain AU01 genome; Supplementary File S2: FASTA file of *P. diazotrophicus* strain AU02 genome; Supplementary File S3: FASTA file of *Kosakonia* sp. strain PdW1; Supplementary File S4: TYGS generated results of the three isolates discussed in this study; Supplementary File S5: Full ASV table with sequences of each ASV used for the microbiome analysis discussed in this study.

## Abbreviations

ANCOM-BC2	Analysis of Compositions of Microbiomes with Bias Correction 2
ANI	Average Nucleotide Identity
ASV	Amplicon Sequence Variants
HSD	High Sensitivity DNA
ONT	Oxford Nanopore Technology
PCoA	Principal Coordinate Analysis
PERMANOVA	Permutational Multivariate Analysis of Variance
PNA	Peptide Nucleic Acid
RPM	Revolutions Per Minute
SDW	Sterile Distilled Water
WGS	Whole Genome Sequencing
