# Supplementary material for: Isolation of N-Fixing Bacteria from Warm-Season Pasture Grasses and the Evaluation of Nitrogen Effects on the Bacterial Communities Present in Cenchrus clandestinus
Source: Microorganisms. 2026 Mar 30;14(4):786. doi: 10.3390/microorganisms14040786 (PMC13118717; doi:10.3390/microorganisms14040786)
Supplement: Supplementary file 1 [file microorganisms-14-00786-s001.zip › SupplementaryData/TYGS_job_results.pdf]

TYPE (STRAIN) GENOME SERVER

JOB SUMMARY

PRINT DATE:	2026-02-17 10:25:32 +0100
JOB ID:	d0ae32b6-17d7-4b3f-b25b-822e5a8a5c48
RESULT PAGE:	https://tygs.dsmz.de/user_results/show?guid=d0ae32b6-17d7-4b3f-b25b-822e5a8a5c48

Table 1: Phylogenies

Publication-ready versions of both the genome-scale GBDP tree and the 16S rRNA gene sequence tree can be customized and exported either in SVG (vector graphic) or PNG format from within the phylogeny viewers in your TYGS result page. For publications the **SVG format is recommended** because it is lossless, always keeps its high resolution and can also be easily converted to other popular formats such as PDF or EPS. Please follow the link provided above!

Table 2: Identification

The below list contains the result of the TYGS species identification routine.

Explanation of remarks that might occur in the below table:

remark [R1]: The TYGS type strain database is automatically updated on an almost daily basis. However, if a particular type strain genome is not available in the TYGS database, this can have several reasons which are detailed in the FAQ. You can request an extended 16S rRNA gene analysis via the 16S tree viewer found in your result page to detect **not yet genome-sequenced** type strains relevant for your study.

remark [R2]: > 70% dDDH value (formula d_4) and (almost) minimal dDDH values for gene-content formulae d_0 and d_6 indicate a potentially unreliable identification result and should thus be checked via the 16S rRNA gene sequence similarity. Such strong deviations can, in principle, be caused by sequence contamination.

remark [R3]: G+C content difference of > 1 % indicates a potentially unreliable identification result because within species G+C content varies no more than 1 %, if computed from genome sequences (PMID: 24505073).

Strain	Conclusion	Identification result	Remark
**'P_diazotrophicus_Strain2_Genome'**	belongs to known species	*Phytobacter diazotrophicus*	
**'P_diazotrophicus_Strain1_Genome'**	belongs to known species	*Phytobacter diazotrophicus*	
**'Kosakonia_sp_Genome'**	potential new species		see [R1]

Table 3: Pairwise comparisons of user genomes vs. type-strain genomes

The following table contains the pairwise dDDH values between your user genomes and the selected type-strain genomes. The dDDH values are provided along with their confidence intervals (C.I.) for the three different GBDP formulas:

- formula d_0 (a.k.a. GGDC formula 1): length of all HSPs divided by total genome length
- formula d_4 (a.k.a. GGDC formula 2): sum of all identities found in HSPs divided by overall HSP length
- formula d_6 (a.k.a. GGDC formula 3): sum of all identities found in HSPs divided by total genome length

Note: Formula d_4 is independent of genome length and is thus robust against the use of incomplete draft genomes. For other reasons for preferring formula d_4, see the FAQ.

Query	Subject	d_0	C.I. d_0	d_4	C.I. d_4	d_6	C.I. d_6	Diff. G+C Percent
**'P_diazotrophicus_Strain 2_Genome.fasta'**	**'P_diazotrophicus_Strain 1_Genome.fasta'**	95.1	[92.8 - 96.7]	95.0	[93.4 - 96.3]	96.8	[95.3 - 97.8]	0.2
**'P_diazotrophicus_Strain 2_Genome.fasta'**	*Phytobacter diazotrophicus* DSM 17806	92.7	[89.9 - 94.8]	93.1	[91.1 - 94.6]	94.9	[93.0 - 96.3]	0.1
**'P_diazotrophicus_Strain 1_Genome.fasta'**	*Phytobacter diazotrophicus* DSM 17806	94.6	[92.1 - 96.3]	92.0	[89.9 - 93.7]	96.1	[94.4 - 97.3]	0.1
**'P_diazotrophicus_Strain 1_Genome.fasta'**	*Citrobacter bitternis* JCM 30009	89.3	[85.9 - 91.9]	91.0	[88.8 - 92.8]	92.1	[89.7 - 94.1]	0.11
**'P_diazotrophicus_Strain 1_Genome.fasta'**	*Kluyvera intestini* GT-16	86.0	[82.3 - 89.0]	91.0	[88.8 - 92.8]	89.6	[86.8 - 91.9]	0.13
**'P_diazotrophicus_Strain 2_Genome.fasta'**	*Kluyvera intestini* GT-16	85.9	[82.3 - 88.9]	90.7	[88.5 - 92.6]	89.5	[86.7 - 91.8]	0.08
**'P_diazotrophicus_Strain 2_Genome.fasta'**	*Citrobacter bitternis* JCM 30009	89.2	[85.8 - 91.8]	90.6	[88.4 - 92.5]	92.0	[89.5 - 93.9]	0.09
**'Kosakonia_sp_Genome.fasta'**	*Kosakonia sacchari* SP1	85.2	[81.4 - 88.2]	68.8	[65.8 - 71.7]	85.1	[81.9 - 87.8]	0.06
**'Kosakonia_sp_Genome.fasta'**	*Kosakonia oryzae* Ola 51	84.0	[80.2 - 87.2]	68.7	[65.7 - 71.6]	84.1	[80.8 - 86.9]	0.2
**'Kosakonia_sp_Genome.fasta'**	*Kosakonia radicincitans* DSM 16656	76.4	[72.5 - 80.0]	63.3	[60.4 - 66.1]	76.5	[73.0 - 79.6]	0.53
**'Kosakonia_sp_Genome.fasta'**	*Kosakonia oryziphila* REICA_142	58.5	[54.9 - 62.1]	53.7	[51.0 - 56.4]	58.6	[55.3 - 61.7]	1.46
**'Kosakonia_sp_Genome.fasta'**	*Kosakonia arachidis* Ah-143	67.5	[63.6 - 71.1]	51.8	[49.1 - 54.4]	65.8	[62.4 - 69.0]	1.67
**'P_diazotrophicus_Strain 2_Genome.fasta'**	*Phytobacter ursingii* ATCC 27989	75.6	[71.6 - 79.2]	45.5	[42.9 - 48.0]	70.3	[66.8 - 73.5]	0.03
**'P_diazotrophicus_Strain 1_Genome.fasta'**	*Phytobacter ursingii* ATCC 27989	76.5	[72.5 - 80.0]	45.4	[42.9 - 48.0]	71.0	[67.5 - 74.2]	0.23
**'P_diazotrophicus_Strain 2_Genome.fasta'**	*Phytobacter palmae* S29	79.7	[75.7 - 83.1]	45.1	[42.5 - 47.6]	73.4	[69.9 - 76.6]	0.37
**'P_diazotrophicus_Strain 1_Genome.fasta'**	*Phytobacter palmae* S29	81.3	[77.4 - 84.6]	45.1	[42.6 - 47.7]	74.7	[71.3 - 77.9]	0.57
**'Kosakonia_sp_Genome.fasta'**	*Kosakonia sacchari* SP1	54.4	[50.9 - 57.9]	28.0	[25.6 - 30.5]	46.3	[43.3 - 49.3]	0.47
**'Kosakonia_sp_Genome.fasta'**	*Kosakonia pseudosacchari* JM-387	53.4	[49.9 - 56.9]	27.9	[25.5 - 30.4]	45.6	[42.6 - 48.6]	0.31
**'Kosakonia_sp_Genome.fasta'**	*Kosakonia sacchari* CGMCC 1.12102	54.1	[50.6 - 57.5]	27.8	[25.5 - 30.3]	46.0	[43.0 - 49.0]	0.46
**'Kosakonia_sp_Genome.fasta'**	*Kosakonia styphnolobii* H02	49.4	[45.9 - 52.8]	27.8	[25.4 - 30.3]	42.7	[39.8 - 45.8]	0.03
**'Kosakonia_sp_Genome.fasta'**	*Kosakonia quasisacchari* WCHEs120001T	54.9	[51.4 - 58.4]	27.6	[25.2 - 30.1]	46.4	[43.4 - 49.5]	0.87

Query	Subject	d_0	C.I. d_0	d_4	C.I. d_4	d_6	C.I. d_6	Diff. G+C Percent
**'Kosakonia_sp_Genome.fasta'**	*Kosakonia oryzendophytica* REICA_082	54.1	[50.6 - 57.5]	26.4	[24.1 - 28.9]	45.2	[42.2 - 48.2]	0.48
**'Kosakonia_sp_Genome.fasta'**	*Kosakonia beeri* AX9bT	49.5	[46.1 - 52.9]	25.8	[23.5 - 28.3]	41.9	[38.9 - 44.9]	0.55
**'P_diazotrophicus_Strain2_Genome.fasta'**	*Phytobacter massiliensis* JC163	37.3	[34.0 - 40.8]	24.6	[22.3 - 27.1]	33.1	[30.1 - 36.1]	2.54
**'P_diazotrophicus_Strain2_Genome.fasta'**	*Phytobacter cepae* AG2aT	50.4	[46.9 - 53.8]	24.6	[22.3 - 27.0]	41.8	[38.8 - 44.8]	0.6
**'P_diazotrophicus_Strain1_Genome.fasta'**	*Phytobacter massiliensis* JC163	37.6	[34.2 - 41.1]	24.6	[22.3 - 27.1]	33.2	[30.3 - 36.3]	2.33
**'P_diazotrophicus_Strain1_Genome.fasta'**	*Phytobacter cepae* AG2aT	50.9	[47.4 - 54.3]	24.6	[22.3 - 27.0]	42.1	[39.1 - 45.1]	0.4
**'P_diazotrophicus_Strain2_Genome.fasta'**	**'Kosakonia_sp_Genome.fasta'**	32.4	[29.1 - 36.0]	23.4	[21.1 - 25.9]	29.2	[26.2 - 32.3]	1.27
**'Kosakonia_sp_Genome.fasta'**	*Phytobacter massiliensis* JC163	29.7	[26.3 - 33.3]	23.4	[21.1 - 25.9]	27.2	[24.3 - 30.3]	1.27
**'Kosakonia_sp_Genome.fasta'**	**'P_diazotrophicus_Strain1_Genome.fasta'**	32.5	[29.2 - 36.1]	23.4	[21.1 - 25.9]	29.2	[26.3 - 32.3]	1.06
**'Kosakonia_sp_Genome.fasta'**	*Kluyvera intestini* GT-16	31.6	[28.2 - 35.2]	23.4	[21.1 - 25.9]	28.6	[25.7 - 31.7]	1.19
**'Kosakonia_sp_Genome.fasta'**	*Phytobacter diazotrophicus* DSM 17806	32.3	[28.9 - 35.9]	23.2	[20.9 - 25.6]	29.0	[26.1 - 32.1]	1.16
**'P_diazotrophicus_Strain2_Genome.fasta'**	*Kosakonia sacchari* SP1	32.6	[29.2 - 36.1]	23.2	[20.9 - 25.7]	29.2	[26.3 - 32.3]	1.32
**'P_diazotrophicus_Strain2_Genome.fasta'**	*Kosakonia oryzae* Ola 51	32.3	[28.9 - 35.9]	23.2	[20.9 - 25.7]	29.0	[26.1 - 32.1]	1.07
**'P_diazotrophicus_Strain1_Genome.fasta'**	*Kosakonia sacchari* SP1	32.9	[29.5 - 36.4]	23.2	[20.9 - 25.7]	29.4	[26.5 - 32.5]	1.12
**'P_diazotrophicus_Strain1_Genome.fasta'**	*Kosakonia oryzae* Ola 51	32.5	[29.1 - 36.1]	23.2	[20.9 - 25.7]	29.2	[26.3 - 32.3]	0.87
**'Kosakonia_sp_Genome.fasta'**	*Phytobacter ursingii* ATCC 27989	32.5	[29.2 - 36.1]	23.1	[20.8 - 25.6]	29.2	[26.2 - 32.3]	1.29
**'P_diazotrophicus_Strain2_Genome.fasta'**	*Kosakonia radicincitans* DSM 16656	31.2	[27.8 - 34.8]	23.1	[20.8 - 25.6]	28.2	[25.3 - 31.3]	0.74
**'P_diazotrophicus_Strain1_Genome.fasta'**	*Kosakonia radicincitans* DSM 16656	31.6	[28.3 - 35.2]	23.1	[20.8 - 25.6]	28.5	[25.6 - 31.6]	0.54
**'Kosakonia_sp_Genome.fasta'**	*Citrobacter bitternis* JCM 30009	31.7	[28.3 - 35.3]	23.1	[20.8 - 25.5]	28.5	[25.6 - 31.6]	1.18
**'P_diazotrophicus_Strain2_Genome.fasta'**	*Kosakonia sacchari* SP1	34.6	[31.3 - 38.2]	23.0	[20.7 - 25.5]	30.6	[27.7 - 33.7]	0.8
**'P_diazotrophicus_Strain1_Genome.fasta'**	*Kosakonia styphnolobii* H02	32.8	[29.4 - 36.3]	23.0	[20.7 - 25.5]	29.3	[26.4 - 32.4]	1.09
**'P_diazotrophicus_Strain2_Genome.fasta'**	*Kosakonia styphnolobii* H02	32.3	[28.9 - 35.9]	23.0	[20.7 - 25.5]	29.0	[26.0 - 32.1]	1.3
**'P_diazotrophicus_Strain2_Genome.fasta'**	*Kosakonia quasisacchari* WCHEs120001T	32.8	[29.4 - 36.4]	22.9	[20.6 - 25.3]	29.3	[26.3 - 32.4]	0.39
**'P_diazotrophicus_Strain1_Genome.fasta'**	*Kosakonia sacchari* SP1	34.2	[30.8 - 37.7]	22.9	[20.6 - 25.4]	30.2	[27.3 - 33.3]	0.6
**'P_diazotrophicus_Strain2_Genome.fasta'**	*Kosakonia pseudosacchari* JM-387	33.5	[30.1 - 37.1]	22.9	[20.6 - 25.3]	29.8	[26.8 - 32.9]	0.95
**'P_diazotrophicus_Strain2_Genome.fasta'**	*Kosakonia oryziphila* REICA_142	30.1	[26.7 - 33.7]	22.8	[20.5 - 25.2]	27.3	[24.4 - 30.4]	0.19
**'P_diazotrophicus_Strain1_Genome.fasta'**	*Kosakonia pseudosacchari* JM-387	34.3	[30.9 - 37.8]	22.8	[20.5 - 25.3]	30.3	[27.3 - 33.4]	0.75

Query	Subject	d_0	C.I. d_0	d_4	C.I. d_4	d_6	C.I. d_6	Diff. G+C Percent
**'P_diazotrophicus_Strain2_Genome.fasta'**	*Kosakonia sacchari* CGMCC 1.12102	34.3	[31.0 - 37.9]	22.8	[20.5 - 25.3]	30.3	[27.4 - 33.4]	0.8
**'P_diazotrophicus_Strain2_Genome.fasta'**	*Kosakonia beeri* AX9bT	32.2	[28.8 - 35.8]	22.7	[20.4 - 25.1]	28.8	[25.9 - 31.9]	0.71
**'P_diazotrophicus_Strain1_Genome.fasta'**	*Kosakonia oryziphila* REICA_142	30.4	[27.0 - 34.0]	22.7	[20.5 - 25.2]	27.5	[24.6 - 30.6]	0.39
**'P_diazotrophicus_Strain1_Genome.fasta'**	*Kosakonia sacchari* CGMCC 1.12102	33.9	[30.5 - 37.5]	22.7	[20.4 - 25.1]	30.0	[27.1 - 33.1]	0.6
**'Kosakonia_sp_Genome.fasta'**	*Phytobacter palmae* S29	32.0	[28.6 - 35.6]	22.7	[20.4 - 25.2]	28.7	[25.8 - 31.8]	1.64
**'P_diazotrophicus_Strain1_Genome.fasta'**	*Kosakonia quasisacchari* WCHEs120001T	32.7	[29.3 - 36.2]	22.7	[20.5 - 25.2]	29.1	[26.2 - 32.3]	0.19
**'P_diazotrophicus_Strain1_Genome.fasta'**	*Kosakonia beeri* AX9bT	32.4	[29.0 - 35.9]	22.7	[20.4 - 25.1]	28.9	[26.0 - 32.0]	0.51
**'Kosakonia_sp_Genome.fasta'**	*Phytobacter cepae* AG2aT	32.2	[28.9 - 35.8]	22.6	[20.3 - 25.0]	28.8	[25.9 - 31.9]	0.67
**'P_diazotrophicus_Strain1_Genome.fasta'**	*Kosakonia arachidis* Ah-143	31.7	[28.3 - 35.3]	22.6	[20.3 - 25.0]	28.4	[25.5 - 31.5]	0.6
**'P_diazotrophicus_Strain2_Genome.fasta'**	*Kosakonia arachidis* Ah-143	31.4	[28.1 - 35.0]	22.6	[20.3 - 25.1]	28.2	[25.3 - 31.3]	0.4
**'P_diazotrophicus_Strain2_Genome.fasta'**	*Kosakonia oryzendophytica* REICA_082	35.3	[31.9 - 38.8]	22.5	[20.2 - 25.0]	30.9	[28.0 - 34.0]	0.78
**'P_diazotrophicus_Strain1_Genome.fasta'**	*Enterobacter cloacae* ATCC 13047	25.8	[22.5 - 29.4]	22.5	[20.3 - 25.0]	24.1	[21.2 - 27.2]	1.44
**'P_diazotrophicus_Strain1_Genome.fasta'**	*Kosakonia oryzendophytica* REICA_082	35.3	[32.0 - 38.9]	22.5	[20.3 - 25.0]	30.9	[28.0 - 34.0]	0.58
**'P_diazotrophicus_Strain2_Genome.fasta'**	*Enterobacter cloacae* ATCC 13047	25.6	[22.3 - 29.3]	22.5	[20.3 - 25.0]	23.9	[21.1 - 27.0]	1.64
**'Kosakonia_sp_Genome.fasta'**	*Leclercia adecarboxylata* NBRC 102595	27.1	[23.8 - 30.7]	22.4	[20.2 - 24.9]	25.0	[22.2 - 28.1]	1.36
**'Kosakonia_sp_Genome.fasta'**	*Enterobacter pasteurii* A-8	28.2	[24.8 - 31.8]	22.4	[20.2 - 24.9]	25.8	[22.9 - 28.9]	2.2
**'Kosakonia_sp_Genome.fasta'**	*Klebsiella quasipneumoniae* 01A030	24.3	[21.0 - 27.9]	22.3	[20.0 - 24.8]	22.9	[20.0 - 25.9]	3.76
**'Kosakonia_sp_Genome.fasta'**	*Enterobacter cloacae* ATCC 13047	29.6	[26.2 - 33.2]	22.3	[20.0 - 24.7]	26.8	[23.9 - 29.9]	0.37
**'Kosakonia_sp_Genome.fasta'**	*Silvania confinis* H4N4	27.3	[24.0 - 31.0]	22.3	[20.0 - 24.7]	25.2	[22.3 - 28.3]	1.47
**'P_diazotrophicus_Strain2_Genome.fasta'**	*Leclercia adecarboxylata* NBRC 102595	25.4	[22.1 - 29.1]	22.2	[19.9 - 24.6]	23.7	[20.9 - 26.8]	2.62
**'P_diazotrophicus_Strain2_Genome.fasta'**	*Enterobacter pasteurii* A-8	25.8	[22.5 - 29.5]	22.2	[19.9 - 24.7]	24.0	[21.2 - 27.1]	3.46
**'Kosakonia_sp_Genome.fasta'**	*Enterobacter timonensis* mt20	29.4	[26.1 - 33.0]	22.2	[19.9 - 24.6]	26.7	[23.8 - 29.8]	2.6
**'P_diazotrophicus_Strain1_Genome.fasta'**	*Enterobacter pasteurii* A-8	25.9	[22.6 - 29.6]	22.2	[19.9 - 24.6]	24.1	[21.2 - 27.2]	3.26
**'P_diazotrophicus_Strain1_Genome.fasta'**	*Silvania confinis* H4N4	26.3	[22.9 - 29.9]	22.1	[19.8 - 24.5]	24.3	[21.5 - 27.4]	2.53
**'P_diazotrophicus_Strain2_Genome.fasta'**	*Silvania confinis* H4N4	26.1	[22.8 - 29.8]	22.1	[19.8 - 24.6]	24.2	[21.4 - 27.3]	2.74
**'P_diazotrophicus_Strain1_Genome.fasta'**	*Enterobacter timonensis* mt20	25.1	[21.8 - 28.7]	22.1	[19.8 - 24.6]	23.4	[20.6 - 26.5]	3.67

Query	Subject	d_0	C.I. d_0	d_4	C.I. d_4	d_6	C.I. d_6	Diff. G+C Percent
**'P_diazotrophicus_Strain 2_Genome.fasta'**	*Enterobacter timonensis* mt20	24.8	[21.5 - 28.5]	22.1	[19.8 - 24.5]	23.2	[20.4 - 26.3]	3.87
**'P_diazotrophicus_Strain 2_Genome.fasta'**	*Klebsiella quasipneumoniae* 01A030	21.9	[18.7 - 25.5]	22.1	[19.8 - 24.5]	21.0	[18.2 - 24.0]	5.03
**'P_diazotrophicus_Strain 1_Genome.fasta'**	*Leclercia adecarboxylata* NBRC 102595	25.6	[22.3 - 29.3]	22.1	[19.9 - 24.6]	23.8	[21.0 - 26.9]	2.42
**'P_diazotrophicus_Strain 1_Genome.fasta'**	*Klebsiella quasipneumoniae* 01A030	22.2	[18.9 - 25.8]	22.0	[19.7 - 24.4]	21.2	[18.4 - 24.3]	4.82
**'Kosakonia_sp_Genome.fasta'**	*Yokenella regensburgei* ATCC 49455	24.3	[21.0 - 28.0]	21.9	[19.7 - 24.4]	22.8	[20.0 - 25.9]	0.56
**'P_diazotrophicus_Strain 2_Genome.fasta'**	*Yokenella regensburgei* ATCC 49455	23.3	[20.0 - 26.9]	21.8	[19.6 - 24.3]	22.0	[19.2 - 25.1]	1.83
**'P_diazotrophicus_Strain 1_Genome.fasta'**	*Yokenella regensburgei* ATCC 49455	23.2	[19.9 - 26.9]	21.8	[19.6 - 24.3]	22.0	[19.2 - 25.0]	1.63
**'Kosakonia_sp_Genome.fasta'**	*Escherichia hermannii* NBRC 105704T	22.3	[19.1 - 26.0]	21.0	[18.8 - 23.4]	21.1	[18.4 - 24.2]	0.14
**'P_diazotrophicus_Strain 2_Genome.fasta'**	*Escherichia hermannii* NBRC 105704T	20.4	[17.2 - 24.1]	20.9	[18.7 - 23.3]	19.7	[16.9 - 22.7]	1.13
**'P_diazotrophicus_Strain 1_Genome.fasta'**	*Escherichia hermannii* NBRC 105704T	20.6	[17.4 - 24.2]	20.9	[18.7 - 23.3]	19.8	[17.0 - 22.8]	0.93

Table 4: Strains in your dataset

Joint dataset of automatically determined closest type strains (if this mode was chosen), manually selected type strains (if selected accordingly) and the provided user strains, if provided (marked in yellow).

Strain	Authority	Other deposits	Synonyms	Base pairs	Percent G+C	No. proteins	Goldstamp	Bioproject accession	Biosample accession	Assembly accession	IMG OID
Kosakonia quasisacchari WCHEs120001T	Wang et al. 2019	NCTC 14272; GDMCC 1.1570	*Kosakonia quasisacchari*	5140 644	53.3	4796		PRJNA524064	SAMN10995740	GCA_004331415	
Kosakonia pseudosacchari JM-387	Kämpfer et al. 2018	CIP 110597; DSM 27151	*Kosakonia pseudosacchari*	4951 392	53.9	4664	Gp0358858	PRJEB10644	SAMEA104072116	GCA_900184035	
Enterobacter pasteurii A-8	Rahi et al. 2024	CIP 103550; ATCC 23355; CCUG 33777; CECT 5075; DSM 26481; NCTC 13380; WDCM 82	*Enterobacter pasteurii*	4810 455	56.4	4376		PRJNA937600	SAMN33411377	GCA_028890245	
Kosakonia arachidis Ah-143	(Madhaiyan et al. 2010) Brady et al. 2013	KCTC 22375; NCIMB 14469; DSM 25165	*Enterobacter arachidis*; *Kosakonia arachidis*	5133 452	52.5	4861	Gp0116591	PRJEB17579	SAMN05192562	GCA_900116535	
Kluyvera intestini GT-16	Tetz and Tetz 2016		*Kluyvera intestini*	5781 767	53.0	5390		PRJNA342563	SAMN05762249	GCA_001856865	
Kosakonia sacchari SP1	(Zhu et al. 2013) Gu et al. 2014	CGMCC 1.12102; DSM 100203; LMG 26783; SP 1	*Enterobacter sacchari*; *Kosakonia sacchari*	5530 608	54.3	5194	Gp0106921	PRJEB16854	SAMN04487787	GCA_900109485	

Strain	Authority	Other deposits	Synonyms	Base pairs	Percent G+C	No. proteins	Goldstamp	Bioproject accession	Biosample accession	Assembly accession	IMG OID
Escherichia hermannii NBRC 105704T	Brenner et al. 1983	CIP 103176; CIP 104946; ATCC 33650; CCUG 15714; DSM 4560; JCM 1473; CDC 980; CDC 980-72; HAMBI 1693; LMG 7867	*Atlantibacter hermannii*; *Escherichia hermannii*	4489 087	54.1	4160	Gp0017730	PRJDB14	SAMD00041803	GCA_000248015	2514752030
Phytobacter palmae S29	Madhaiyan et al. 2020	DSM 27342; KACC 17598	*Phytobacter palmae*	5283 934	52.6	4972	Gp0116592	PRJEB17238	SAMN05216563	GCA_900112785	
Klebsiella quasipneumoniae 01A030	Brisse et al. 2014	CIP 110771; DSM 28211; SB11	*Klebsiella quasipneumoniae*; *Klebsiella quasipneumoniae* subsp. *quasipneumoniae*	5457 795	58.0	5287	Gp0102348	PRJEB6037	SAMEA2471851	GCA_000751755	
Kosakonia styphnolobii H02	Xu et al. 2026	KCTC 8162; GDMCC 1.4158	*Kosakonia styphnolobii*	4439 479	54.2	4048		PRJNA999061	SAMN36720165	GCA_030704225	
Phytobacter cepae AG2aT	Jordan et al. 2026	CCOS 2093; CFBP 9466	*Phytobacter cepae*	5158 755	53.5	4724		PRJNA1310503	SAMN50772893	GCA_054165735	
Kosakonia beeri AX9bT	Jordan et al. 2026	CCOS 2091; CFBP 9467	*Kosakonia beeri*	4921 244	53.7	4528		PRJNA1310503	SAMN50772894	GCA_054165705	
Kosakonia radicincitans DSM 16656	(Kämpfer et al. 2005) Brady et al. 2013	CIP 108468; CCUG 50898; D5/23	*Enterobacter radicincitans*; *Kosakonia radicincitans*	6041 571	53.7	6135	Gp0021125	PRJNA161109	SAMN02470245	GCA_000280495	2531839681

Strain	Authority	Other deposits	Synonyms	Base pairs	Percent G+C	No. proteins	Goldstamp	Bioproject accession	Biosample accession	Assembly accession	IMG OID
Yokenella regensburgei ATCC 49455	Kosako et al. 1985	BCRC 12225; CCRC 12225; CIP 105435; JCM 2403; NBRC 102600; NCTC 11966; NIH 725-83	*Yokenella regensburgei*	4853 439	54.8	4564	Gp0009521	PRJNA59563	SAMN02743390	GCA_000735455	
Leclercia adecarboxylata NBRC 102595	(Leclerc 1962) Tamura et al. 1987 emend. Maddock et al. 2022	CIP 82.92; ATCC 23216; JCM 1667; NCTC 13032; HAMBI 1696; LMG 2803	*Escherichia adecarboxylata*; *Leclercia adecarboxylata*	4991 157	55.6	4630	Gp0009485	PRJNA59559	SAMN02743265	GCA_000735515	
Kosakonia oryzae Ola 51	(Peng et al. 2009) Brady et al. 2013	CGMCC 1.7012; LMG 24251	*Enterobacter oryzae*; *Kosakonia oryzae*	5303 342	54.0	4773	Gp0154734	PRJNA309028	SAMN04419549	GCA_001658025	
Kosakonia oryzendophytica REICA_082	(Hardoim et al. 2015) Li et al. 2016	NCCB 100390; LMG 26432	*Enterobacter oryzendophyticus*; *Kosakonia oryzendophytica*	4837 282	53.7	4486	Gp0108252	PRJEB15027	SAMN04487791	GCA_900094925	
Kosakonia oryziphila REICA_142	(Hardoim et al. 2015) Li et al. 2016	NCCB 100393; LMG 26429	*Enterobacter oryziphilus*; *Kosakonia oryziphila*	4811 075	52.8	4667	Gp0108251	PRJEB15030	SAMN04487790	GCA_900094795	
Phytobacter massiliensis JC163	(Lagier et al. 2014) Ma et al. 2021	DSM 26120; CSUR P161	*Enterobacter massiliensis*; *Metakosakonia massiliensis*; *Phytobacter massiliensis*	4922 242	55.5	4651	Gp0012776	PRJEA70551	SAMEA2272705	GCA_000321045	2547132115
Citrobacter bitternis JCM 30009	Ko et al. 2015	KCTC 42139; SKKU-TP7	*Citrobacter bitternis*	5644 558	53.0	5311		PRJNA595761	SAMN43284229	GCA_042658825	
Kosakonia sacchari CGMCC 1.12102	(Zhu et al. 2013) Gu et al. 2014	CGMCC 1.12102; DSM 100203; LMG 26783; SP 1	*Enterobacter sacchari*; *Kosakonia sacchari*	4858 922	53.7	4537	Gp0093897	PRJEB16027	SAMN02927897	GCA_900100995	

Strain	Authority	Other deposits	Synonyms	Base pairs	Percent G+C	No. proteins	Goldstamp	Bioproject accession	Biosample accession	Assembly accession	IMG OID
Kosakonia sacchari SP1	(Zhu et al. 2013) Gu et al. 2014	CGMCC 1.12102; DSM 100203; LMG 26783; SP 1	*Enterobacter sacchari*; *Kosakonia sacchari*	4902 027	53.7	4581	Gp0033762	PRJNA224116	SAMN02472168	GCF_000300455	2547132181
Enterobacter cloacae ATCC 13047	(Jordan 1890) Hormaeche and Edwards 1960	CIP 60.85; CCUG 28448; CCUG 29301; CCUG 6323; DSM 30054; JCM 1232; NBIMCC 8570; IFO 13535; NBRC 13535; NCTC 10005; HAMBI 1295; HAMBI 96; LMG 2783	*Bacillus cloacae*; *Bacterium cloacae*; *Cloaca cloacae*; *Enterobacter cloacae*; *Enterobacter cloacae* subsp. *cloacae*	5598 795	54.6	5518	Gp0006802	PRJNA45793	SAMN02603901	GCA_000025565	646564529
Phytobacter diazotrophicus DSM 17806	Zhang et al. 2017 emend. Pillonetto et al. 2018	CGMCC 1.5339; CGMCC 1.5539; LMG 23328; LS 8	*Phytobacter diazotrophicus*	5540 150	53.0	5199	Gp0290569	PRJNA500337	SAMN10362891	GCA_004346725	2784746789
Phytobacter ursingii ATCC 27989	Pillonetto et al. 2018	CNCTC 5729	*Phytobacter ursingii*	5755 402	52.9	5450		PRJEB32294	SAMEA5577247	GCA_901456055	
Enterobacter timonensis mt20	Takakura et al. 2019	CSUR P2201	*Enterobacter timonensis*	4199 688	56.8	3851		PRJEB12555	SAMEA3859023	GCA_900021175	
Silvania confinis H4N4	Maddock et al. 2023	CCUG 76175; CCUG T 76175; LMG 32607	*Silvania confinis*	4864 404	55.7	4524		PRJNA837589	SAMN28207119	GCA_025564085	
P_diazotrophicus_Strain2_Genome.fasta				5631 480	52.9	5281					

Strain	Authority	Other deposits	Synonyms	Base pairs	Percent G+C	No. proteins	Goldstamp	Bioproject accession	Biosample accession	Assembly accession	IMG OID
Kosakonia_sp_Genome.fasta				5635325	54.2	5242					
P_diazotrophicus_Strain1_Genome.fasta				5586165	53.1	5228					

Methods, Results and References

The genome sequence data were uploaded to the Type (Strain) Genome Server (TYGS), a free bioinformatics platform available under https://tygs.dsmz.de, for a whole genome-based taxonomic analysis [1]. The analysis also made use of recently introduced methodological updates and features [2,3]. Information on nomenclature, synonymy and associated taxonomic literature was provided by TYGS's sister database, the List of Prokaryotic names with Standing in Nomenclature (LPSN, available at https://lpsn.dsmz.de) [2,3]. The results were provided by the TYGS on 2026-02-17. The TYGS analysis was subdivided into the following steps:

Determination of closely related type strains

Determination of closest type strain genomes was done in two complementary ways: First, all user genomes were compared against all type strain genomes available in the TYGS database via the MASH algorithm, a fast approximation of intergenomic relatedness [4], and, the ten type strains with the smallest MASH distances chosen per user genome. Second, an additional set of ten closely related type strains was determined via the 16S rDNA gene sequences. These were extracted from the user genomes using RNAmmer [5] and each sequence was subsequently BLASTed [6] against the 16S rDNA gene sequence of each of the currently 24127 type strains available in the TYGS database. This was used as a proxy to find the best 50 matching type strains (according to the bitscore) for each user genome and to subsequently calculate precise distances using the Genome BLAST Distance Phylogeny approach (GBDP) under the algorithm 'coverage' and distance formula d_5 [7]. These distances were finally used to determine the 10 closest type strain genomes for each of the user genomes.

Pairwise comparison of genome sequences

For the phylogenomic inference, all pairwise comparisons among the set of genomes were conducted using GBDP and accurate intergenomic distances inferred under the algorithm 'trimming' and distance formula d_5 [7]. 100 distance replicates were calculated each. Digital DDH values and confidence intervals were calculated using the recommended settings of the GGDC 4.0 [2,7].

Phylogenetic inference

The resulting intergenomic distances were used to infer a balanced minimum evolution tree with branch support via FASTME 2.1.6.1 including SPR postprocessing [8]. Branch support was inferred from 100 pseudo-bootstrap replicates each. The trees were rooted at the midpoint [9] and visualized with PhyD3 [10].

Type-based species and subspecies clustering

The type-based species clustering using a 70% dDDH radius around each of the 27 type strains was done as previously described [1]. The resulting groups are shown in Table 1 and 4. Subspecies clustering was done using a 79% dDDH threshold as previously introduced [11].

Results

Type-based species and subspecies clustering

The resulting species and subspecies clusters are listed in Table 4, whereas the taxonomic identification of the query strains is found in Table 1. Briefly, the clustering yielded 24 species clusters and the provided query strains were assigned to 2 of these. Moreover, user strains were located in 2 of 24 subspecies clusters.

Figure caption SSU tree

Figure 1. Tree inferred with FastME 2.1.6.1 [8] from GBDP distances calculated from 16S rDNA gene sequences. The branch lengths are scaled in terms of GBDP distance formula d_5. The numbers above branches are GBDP pseudo-bootstrap support values > 60 % from 100 replications, with an average branch support of 56.3 %. The tree was rooted at the midpoint [9].

Figure caption genome tree

Figure 2. Tree inferred with FastME 2.1.6.1 [8] from GBDP distances calculated from genome sequences. The branch lengths are scaled in terms of GBDP distance formula d_5. The numbers above branches are GBDP pseudo-bootstrap support values > 60 % from 100 replications, with an average branch support of 88.7 %. The tree was rooted at the midpoint [9].

References

[1] Meier-Kolthoff JP, Göker M. TYGS is an automated high-throughput platform for state-of-the-art genome-based taxonomy. Nat. Commun. 2019;10: 2182. DOI: 10.1038/s41467-019-10210-3

[2] Meier-Kolthoff JP, Sardà Carbasse J, Peinado-Olarte RL, Göker M. TYGS and LPSN: a database tandem for fast and reliable genome-based classification and nomenclature of prokaryotes. Nucleic Acid Res. 2022;50: D801–D807. DOI: 10.1093/nar/gkab902

[3] Freese HM, Meier-Kolthoff JP, Sardà Carbasse J, Afolayan AO, Göker M. TYGS and LPSN in 2025: a Global Core Biodata Resource for genome-based classification and nomenclature of prokaryotes within DSMZ Digital Diversity. Nucleic Acid Res. 2025, gkaf1110. DOI: 10.1093/nar/gkaf1110

[4] Ondov BD, Treangen TJ, Melsted P, et al. Mash: Fast genome and metagenome distance estimation using MinHash. Genome Biol 2016;17: 1–14. DOI: 10.1186/s13059-016-0997-x

[5] Lagesen K, Hallin P. RNAmmer: consistent and rapid annotation of ribosomal RNA genes. Nucleic Acids Res. Oxford Univ Press; 2007;35: 3100–3108. DOI: 10.1093/nar/gkm160

[6] Camacho C, Coulouris G, Avagyan V, Ma N, Papadopoulos J, Bealer K, et al. BLAST+: architecture and applications. BMC Bioinformatics. 2009;10: 421. DOI: 10.1186/1471-2105-10-421

[7] Meier-Kolthoff JP, Auch AF, Klenk H-P, Göker M. Genome sequence-based species delimitation with confidence intervals and improved distance functions. BMC Bioinformatics. 2013;14: 60. DOI: 10.1186/1471-2105-14-60

[8] Lefort V, Desper R, Gascuel O. FastME 2.0: A comprehensive, accurate, and fast distance-based phylogeny inference program. Mol Biol Evol. 2015;32: 2798–2800. DOI: 10.1093/molbev/msv150

[9] Farris JS. Estimating phylogenetic trees from distance matrices. Am Nat. 1972;106: 645–667.

[10] Kreft L, Botzki A, Coppens F, Vandepoele K, Van Bel M. PhyD3: A phylogenetic tree viewer with extended phyloXML support for functional genomics data visualization. Bioinformatics. 2017;33: 2946–2947. DOI: 10.1093/bioinformatics/btx324

[11] Meier-Kolthoff JP, Hahnke RL, Petersen J, Scheuner C, Michael V, Fiebig A, et al. Complete genome sequence of DSM 30083^{T}, the type strain (U5/41^{T}) of *Escherichia coli*, and a proposal for delineating subspecies in microbial taxonomy. Stand Genomic Sci. 2014;9: 2. DOI: 10.1186/1944-3277-9-2
